# Identification of Oxidation State +1 in a Molecular Uranium Complex

**DOI:** 10.1021/jacs.2c06519

**Published:** 2022-09-28

**Authors:** Luciano Barluzzi, Sean R. Giblin, Akseli Mansikkamäki, Richard A. Layfield

**Affiliations:** † Department of Chemistry, School of Life Sciences, University of Sussex, Brighton BN1 9JQ, U.K.; ‡ School of Physics and Astronomy, Cardiff University, Cardiff CF24 3AA, U.K.; § NMR Research Group, University of Oulu, P.O. Box 8000, FI-90014 Oulu, Finland

## Abstract

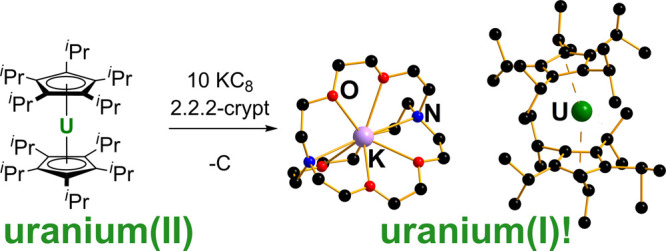

The concept of oxidation state plays a fundamentally important role in defining the chemistry of the elements. In the f block of the periodic table, well-known oxidation states in compounds of the lanthanides include 0, +2, +3 and +4, and oxidation states for the actinides range from +7 to +2. Oxidation state +1 is conspicuous by its absence from the f-block elements. Here we show that the uranium(II) metallocene [U(η^5^-C_5_^*i*^Pr_5_)_2_] and the uranium(III) metallocene [IU(η^5^-C_5_^*i*^Pr_5_)_2_] can be reduced by potassium graphite in the presence of 2.2.2-cryptand to the uranium(I) metallocene [U(η^5^-C_5_^*i*^Pr_5_)_2_]^−^ (**1**) (C_5_^*i*^Pr_5_ = pentaisopropylcyclopentadienyl) as the salt of [K(2.2.2-cryptand)]^+^. An X-ray crystallographic study revealed that **1** has a bent metallocene structure, and theoretical studies and magnetic measurements confirmed that the electronic ground state of uranium(I) adopts a 5f^3^(7s/6d_*z*^2^_)^1^(6d_*x*^2^–*y*^2^_/6d_*xy*_)^1^ configuration. The metal–ligand bonding in **1** consists of contributions from uranium 5f, 6d, and 7s orbitals, with the 6d orbitals engaging in weak but non-negligible covalent interactions. Identification of the oxidation state +1 for uranium expands the range of isolable oxidation states for the f-block elements and potentially signposts a synthetic route to this elusive species for other actinides and the lanthanides.

The oxidation state of an element strongly influences the stability, reactivity, and physical properties of the compounds it forms. There is considerable motivation for isolating elements in new oxidation states since this can lead to new chemistry while also providing a deeper fundamental understanding of bonding and electronic structure. In the lanthanide series, the oxidation state +3 is thermodynamically the most stable species by far. Recent reports of molecular compounds containing praseodymium^1^ and terbium^2−4^ in the oxidation state +4 are therefore notable advances. Similarly, the synthesis and isolation of compounds containing the full series of lanthanides (except promethium) in the oxidation state +2 is a significant achievement^5−7^ and builds on the earlier discovery of formally lanthanide(0) sandwich compounds.^8,9^ In the actinide series, oxidation states range from as high as +7 in the neptunyl cation^10^ to, most recently, +2 in molecular compounds of thorium,^11^ uranium,^12−14^ neptunium,^15^ and plutonium.^16^

We recently reported the linear uranium(II) metallocene [U(η^5^-C_5_^*i*^Pr_5_)_2_], in which uranium has an electron configuration consisting of a 5f^3^ component and an electron residing in a 6d_*z*^2^_/7s hybrid orbital.^17^ A cyclic voltammetry study of this uranocene revealed two electrochemical events, assigned to the uranium(II)/uranium(III) and uranium(III)/uranium(IV) couples. Unexpectedly, further reduction to an unstable species was tentatively attributed to a complex of uranium(I). Notably, no molecular compound of an f-block element in the oxidation state +1 has been isolated. Lanthanum monoiodide (LaI) is best described as a nominally lanthanum(I)-containing extended solid, with metallic properties arising from two electrons per La^+^ donated to the conduction band.^18,19^ A uranium(II) complex that by virtue of ligand noninnocence reacts as a uranium(I) “synthon” was also recently described,^20^ and the formally uranium(I)-containing complex [UFe(CO)_3_]^−^ was detected in the gas phase.^21^ Beyond these examples, a theoretical study has predicted that well-defined uranium(I) compounds should be stable and therefore synthetically accessible from a suitable precursor.^22^

We attempted the reduction of [U(η^5^-C_5_^*i*^Pr_5_)_2_] in hexane using an excess of potassium graphite (KC_8_) and 1 equiv of 2.2.2-cryptand. Over 3 days, the initial green color faded, and a brown solid precipitated. Extraction of the solid into benzene followed by recrystallization produced brown crystals, which X-ray crystallography revealed to be the uranium(I) complex [U(η^5^-C_5_^*i*^Pr_5_)_2_]^−^ (**1**) as the salt of [K(2.2.2-crypt)]^+^ (Scheme 1). Compound [K(2.2.2-crypt)][**1**] was also synthesized by reducing the iodo-ligated uranium(III) metallocene [IU(η^5^-C_5_^*i*^Pr_5_)_2_] under the same conditions. Isolated yields of crystalline material were 55–60%. Reduction of [U(η^5^-C_5_^*i*^Pr_5_)_2_] and [IU(η^5^-C_5_^*i*^Pr_5_)_2_] does not occur in the absence of cryptand.

**Scheme 1:**
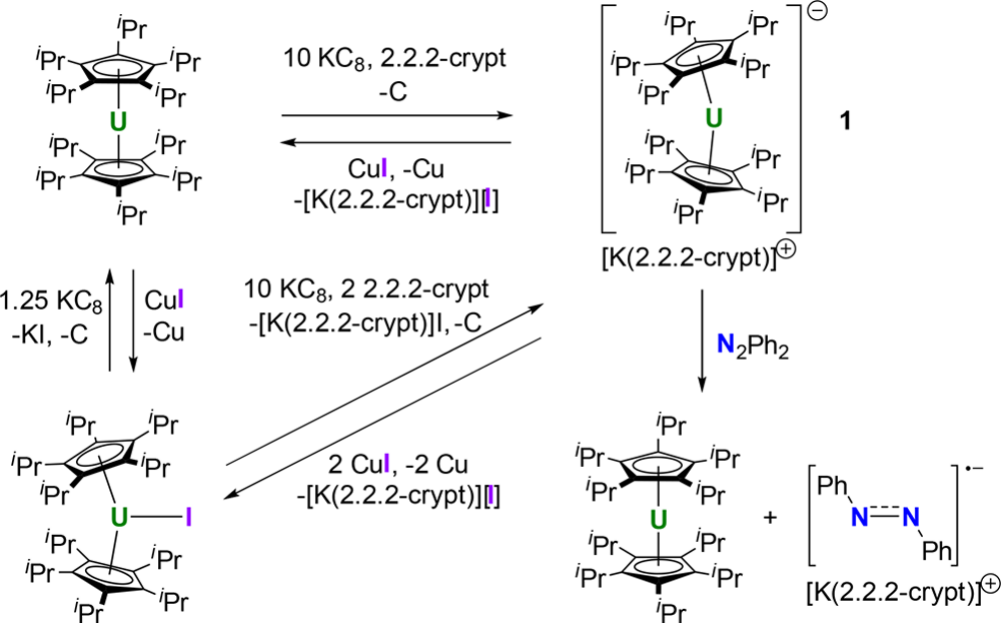
Synthesis of [K(2.2.2-crypt)][**1**] and Its Oxidation with Copper Iodide or Azobenzene

The molecular structure of [K(2.2.2-crypt)][**1**] features two disordered units of **1**. The major component of **1** adopts a bent metallocene geometry with a Cp_cent_–U–Cp_cent_ angle of 163.4(3)° and U–Cp_cent_ distances of 2.564(8) and 2.585(7) Å (Cp_cent_ denotes the center of the pentaisopropylcyclopentadienyl ligands) (Figures 1 and S1 and Table S1). Compared to the linear uranium(II) metallocene [U(η^5^-C_5_^*i*^Pr_5_)_2_], appreciable distortion of the structure occurs upon one-electron reduction to **1**. The U–Cp_cent_ distances in **1** are also significantly longer than the analogous distances of 2.504(1) Å in [U(η^5^-C_5_^*i*^Pr_5_)_2_] and 2.496(3) Å in the uranium(III) complex [U(η^5^-C_5_^*i*^Pr_5_)_2_]^+^,^23^ consistent with the lower uranium oxidation state in **1**.

**Figure 1:**
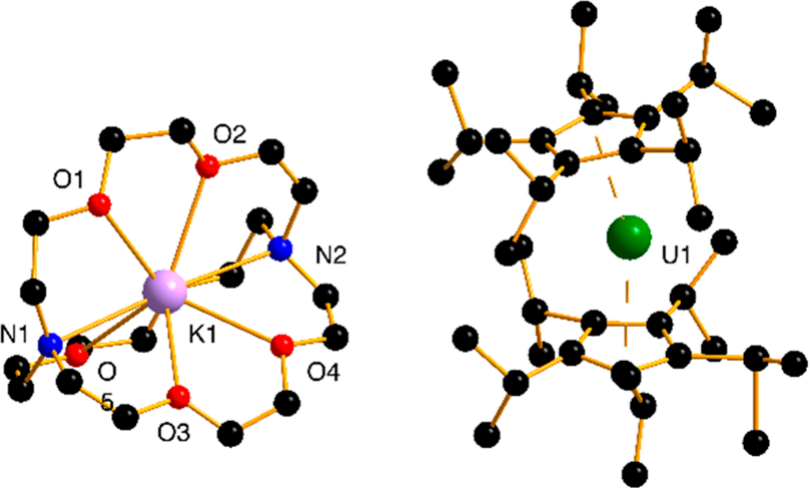
Molecular structure of [K(2.2.2-crypt)][**1**]. For clarity, the carbon atoms in black are not numbered, and the hydrogen atoms are not shown.

Compound [K(2.2.2-crypt)][**1**] can be stored as a solid at −40 °C seemingly indefinitely without decomposition. In contrast, gradual decomposition to a light-brown solid occurs within a few hours at room temperature. Brown solutions of [K(2.2.2-crypt)][**1**] in benzene-*d*_6_ decompose over 5 days to give a green solution that ^1^H NMR spectroscopy revealed to consist of [U(η^5^-C_5_^*i*^Pr_5_)_2_], [K(2.2.2-crypt)][C_5_^*i*^Pr_5_], and a gray precipitate, presumed to be metallic uranium. These observations suggest that the uranium(I) metallocene disproportionates in solution (Figures S3–S5). In THF, [K(2.2.2-crypt)][**1**] decomposes immediately to give an intractable mixture (Figures S6 and S7). To discount the possibility of hydride ligands bound to uranium, carbon tetrachloride was added to [K(2.2.2-crypt)][**1**]. The formation of chloroform or dichloromethane was not observed by ^1^H and ^13^C NMR spectroscopy (Figures S8 and S9). Chemical reversibility of the reactions that form [K(2.2.2-crypt)][**1**] was confirmed by the addition of 1 or 2 equiv of the mild oxidant copper(I) iodide, which led to the formation of [U(η^5^-C_5_^*i*^Pr_5_)_2_] and [IU(η^5^-C_5_^*i*^Pr_5_)_2_], respectively (Scheme 1 and Figures S10 and S11).

To obtain further insight into the electron configuration and bonding in **1**, density functional theory (DFT) calculations were carried out. The electron configuration was determined to be 5f^3^(7s/6d_*z*^2^_)^1^(6d_*x*^2^–y^2^_/6d_*xy*_)^1^. Three electrons occupy orbitals with strong atomic-like 5f character. One electron occupies a quasi-σ-symmetric orbital that is an admixture of the 7s and 6d_*z*^2^_ atomic orbitals. Based on decomposition of the orbital into a basis of uranium(I) orbitals, the orbital has 63% 7s character and 28% 6d character. The orbital has a toroidal shape that is typical for lanthanide(II) and uranium(II) metallocenes.^17,24,25^ The one remaining electron occupies a quasi-δ-symmetric 6d_*x*^2^–*y*^2^_ and 6d_*xy*_ set of orbitals with significant delocalization into the ligands. Several calculations were carried out to see whether a low-lying lower-spin electronic configuration existed, but all lower-spin states discovered lie at higher energy than the highest-spin state.

The bonding in **1** is shown in Figure 2, with quantitative contributions of the uranium and cyclopentadienyl orbitals to the molecular orbitals (MOs) provided in Tables S2 and S3. The occupied 5f, 6d, and 7s orbitals form an energetically closely packed manifold. The three 5f orbitals occupied by three unpaired electrons all have more than 91% 5f character and show very little covalency. The 6d orbitals are weakly mixed with the nearly doubly degenerate highest-occupied MOs of the ligands. The 6d contribution in the main metal–ligand bonding orbitals varies from 0 to 14% depending on the orbital and is evidence of weak but non-negligible uranium–cyclopentadienyl covalency in **1**.

**Figure 2:**
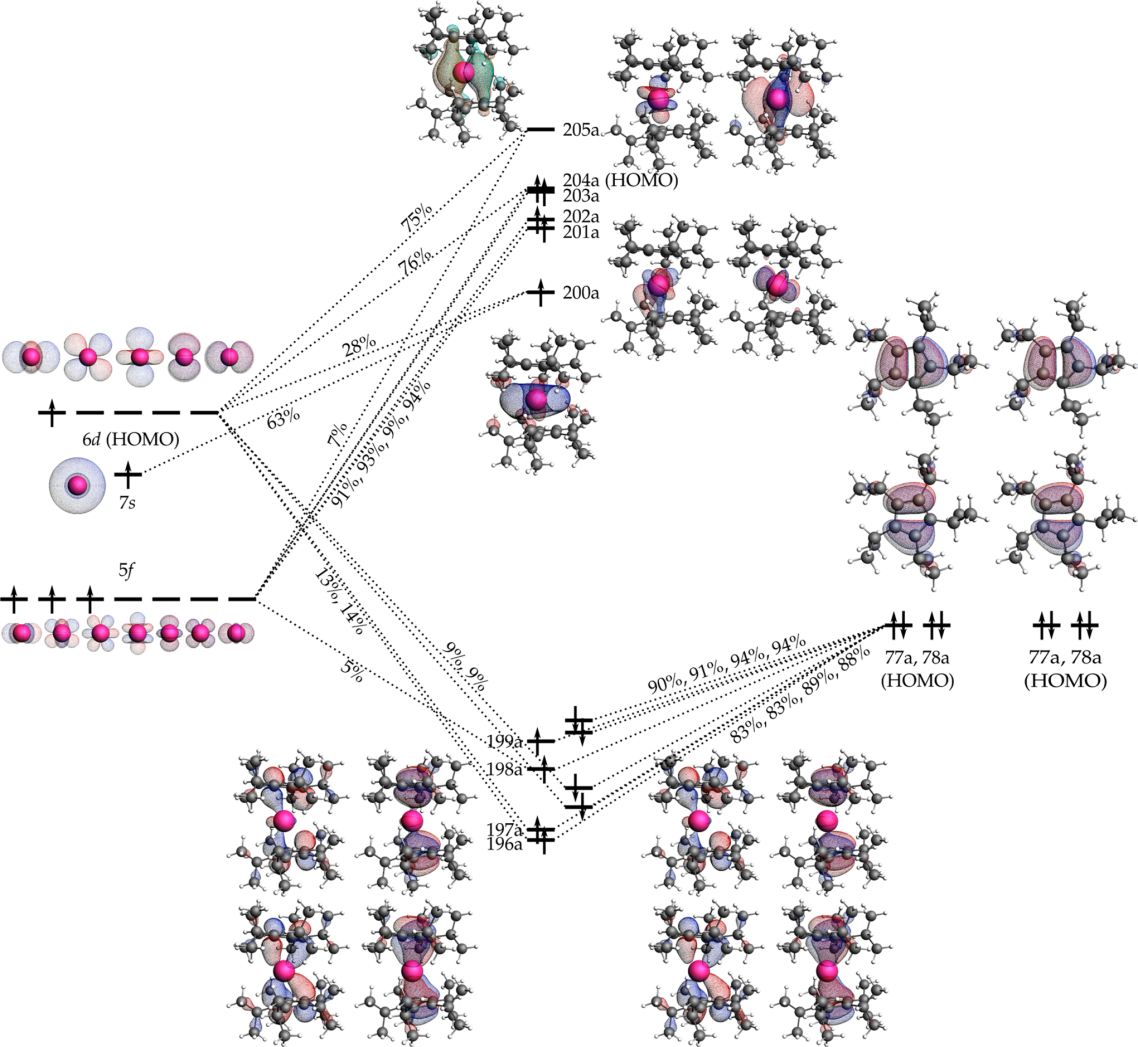
Qualitative molecular orbital diagram for **1**. The numbers given are percentage contributions of the nonorthogonal fragment orbitals to the molecular orbitals. Only contributions larger than 5% are shown.

The molar magnetic susceptibility (χ_M_) of an unrestrained polycrystalline sample of [K(2.2.2-crypt)][**1**] was measured from 2.5 to 200 K in a direct current (dc) field of 1 kOe (Figure 3, left). Above 90 K, χ_M_*T* is strongly temperature-dependent, reaching a value 3.43 cm^3^ K mol^–1^ at 200 K, equivalent to an effective magnetic moment (μ_eff_) of 5.35μ_B_. This magnetic moment is much larger than any reported value for a molecular uranium complex even at 300 K,^26^ including those for [U(η^5^-C_5_^*i*^Pr_5_)_2_]^17^ and [IU(η^5^-C_5_^*i*^Pr_5_)_2_].^23^ Between 90 and 10 K, χ_M_*T* varies only slightly in the range of 0.98–1.17 cm^3^ K mol^–1^ or 2.80–2.90μ_B_ before decreasing sharply to 0.43 cm^3^ K mol^–1^ or 1.85μ_B_ at 2.5 K. The temperature-dependence of χ_M_*T* is unusual and suggests gradual population of a thermally accessible excited electronic state at higher temperatures. At lower temperatures, the sharp drop in χ_M_*T* is consistent with the onset of single-molecule magnet (SMM) behavior. Interpretation of the susceptibility is difficult because of the large number of low-lying states in the uranium(I) ion involving strong interactions of the 7s and 6d orbitals with the ligands.^27^ This leads to a densely packed manifold of thermally accessible states with largely varying magnetic properties. The low-temperature susceptibility can be interpreted in terms of a coupling model where the intershell exchange coupling is stronger than the intrashell *LS* coupling (where *L* and *S* are the orbital and spin angular momenta, respectively; Table S4). The increase in the susceptibility at higher temperatures most likely results from population of thermally accessible states where the spin and orbital momenta are not coupled fully antiparallel, leading to a larger total momentum.

**Figure 3:**
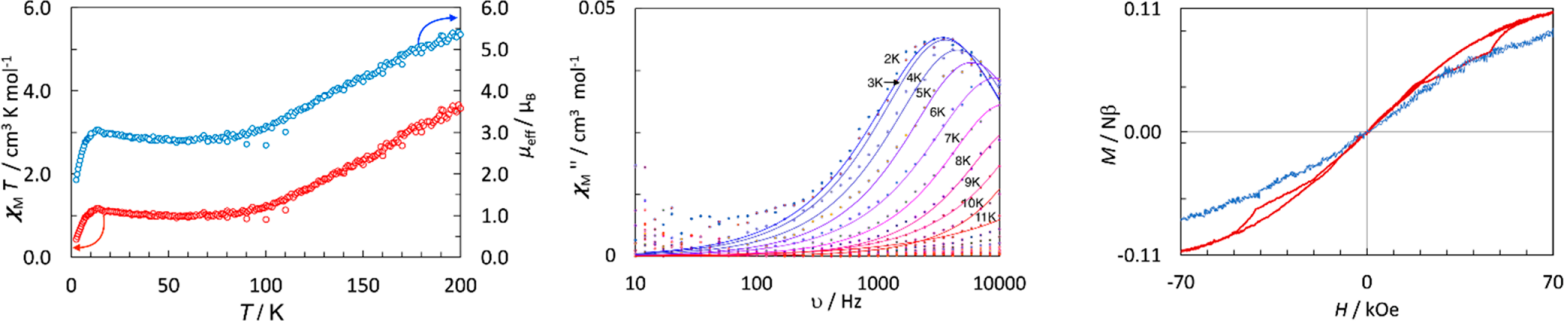
(left) Temperature dependences of χ_M_*T* and μ_eff_ at *T* = 2.5–200 K in a field of 1 kOe. (center) Frequency dependence of χ″ at the temperatures indicated in zero dc field. (right) Hysteresis at 2 K for the unrestrained (red) and restrained (blue) materials using an average sweep rate of 20 Oe s^–1^.

Alternating current (ac) magnetic susceptibility measurements on unrestrained [K(2.2.2-crypt)][**1**] in zero dc field revealed slow relaxation of the magnetization and SMM properties. The imaginary component of the ac susceptibility (χ″) shows frequency (ν)-dependent maxima at *T* = 2.3–8 K (Figure 3 center and Figures S12 and S13). The relaxation times (τ) were extracted from these data, and the temperature dependence of τ was fitted using τ^–1^ = τ_0_^–1^ exp(−*U*_eff_/*k*_B_*T*) (i.e., only an Orbach term), which yielded an effective energy barrier (*U*_eff_) of 14(1) cm^–1^ and a pre-exponential term (τ_0_) of 1.1(3) × 10^–7^ s (Figure S14).

The field dependence of the magnetization was measured for [K(2.2.2-crypt)][**1**] at 2 K on both the unrestrained material and the material restrained in glass wool. In fields below approximately 20 kOe, the two data sets are similar, with magnetic torque effects becoming evident only at higher fields. For the unrestrained sample, magnetic memory effects and very narrow hysteresis loops were observed (Figure 3, right). This behavior is reminiscent of the neptunium(IV) compound neptunocene or [Np(η^8^-COT)_2_] (COT = cyclooctatetraenyl).^28^

The 5f^3^(7s/6d_*z*^2^_)^1^(6d_*x*^2^–*y*^2^_ /6d_*xy*_)^1^ configuration of uranium(I) in [K(2.2.2-crypt)][**1**] produced a rhombic powder X-band EPR spectrum at 10 K with *g* factors of 4.7, 1.6, and 1.2 (Figure S15). In contrast, the non-Kramers uranium(II) center in [U(η^5^-C_5_^*i*^Pr_5_)_2_] is EPR-silent (Figure S16). The UV/vis/NIR spectrum of [K(2.2.2-crypt)][**1**] in benzene is essentially featureless except for intense charge transfer absorptions in the UV region and weak absorptions from 800 to 1450 nm (Figures S17 and S18).

Since complexes of uranium in low oxidation states can be multielectron donors toward small molecules,^29^ we were interested to see how [K(2.2.2-crypt)][**1**] would behave toward azobenzene, N_2_Ph_2_. Adding 1 equiv of azobenzene to [K(2.2.2-crypt)][**1**] in benzene caused a color change from brown to green and precipitation of a dark-brown solid (Scheme 1). ^1^H NMR spectroscopy revealed the formation of [U(η^5^-C_5_^*i*^Pr_5_)_2_] (Figures S19 and S20), suggesting that a one-electron transfer process had occurred. The precipitate was identified by X-ray crystallography and IR and EPR spectroscopies to be the azobenzene radical anion [N_2_Ph_2_]^−^ as the salt of [K(2.2.2-crypt)]^+^ (Table S1 and Figures S21–S24). This one-electron reduction process contrasts to that shown by the uranium(II) complex [U{N(SiMe_3_)_2_}_3_]^−^ toward azobenzene, which initiates a four-electron reduction and cleavage of the nitrogen–nitrogen double bond in the substrate.^30^ This reactivity, combined with the disproportionation of [K(2.2.2-crypt)][**1**] into its uranium(II) precursor and uranium metal, points to unexpected stability of [U(η^5^-C_5_^*i*^Pr_5_)_2_], most likely due to the stabilizing effect of the bulky ligands.

In conclusion, we have shown that a metallocene of uranium in the oxidation state +1 can be synthesized by reduction of uranium(II) and uranium(III) precursors. Reactivity studies, magnetic and spectroscopic measurements, and a DFT study are consistent with the presence of uranium(I) with a 5f^3^(7s/6d_*z*^2^_)^1^(6d_*x*^2^–*y*^2^_ /6d_*xy*_)^1^ ground-state electron configuration. The broader significance of [K(2.2.2-crypt)][**1**] is that soluble molecular compounds of other actinides and some lanthanides in the oxidation state +1 should also be viable targets. The synthesis of a much larger family of compounds containing lanthanide(I) and actinide(I) centers introduces new possibilities for designing molecular magnets and luminescent materials and for developing new f-element reactivity.

## Notes

Additional research data supporting this publication are available at 10.25377/sussex.21184705.

## Supporting Information Available

Synthesis, spectroscopic characterization, crystallographic details, magnetic property measurements, and computational details (PDF)
