# Supplementary material for: Identification of Oxidation State +1 in a Molecular Uranium Complex
Source: J Am Chem Soc. 2022 Sep 28;144(40):18229–33. doi: 10.1021/jacs.2c06519 (PMC9562434; doi:10.1021/jacs.2c06519)
Supplement: Supplementary file 1 — ja2c06519_si_001.pdf [file ja2c06519_si_001.pdf]

Supporting Information for

# Identification of oxidation state +1 in a molecular uranium complex

Luciano Barluzzi,[1] Sean R. Giblin,[2] Akseli Mansikkamäki,[3]* Richard A. Layfield[1]*

1. Department of Chemistry, School of Life Sciences, University of Sussex; Brighton, BN1 9JQ, U.K.
2. School of Physics and Astronomy, Cardiff University; Cardiff, CF24 3AA, U.K.
3. NMR Research Group, University of Oulu; P.O. Box 8000, Oulu FI-90014, Finland.

## General Considerations

The manipulations described below were conducted under argon or nitrogen with rigorous exclusion of air and water (< 0.1 ppm) using an MBraun glovebox and standard Schlenk line techniques. Glassware was dried at 150°C before use. Solvents were purchased from Merck, refluxed over an appropriate drying agent for a minimum of three days (molten K for benzene, $C_6D_6$, THF-$d_8$, and THF; Na/K alloy for hexane and pentane, $CaH_2$ for $CCl_4$) and then distilled, degassed by three cycles of freeze-pump-thawing, and stored in ampoules over potassium mirrors (benzene, hexane, pentane) or 4 Å molecular sieves ($C_6D_6$, THF-$d_8$, THF, $CCl_4$). Unless otherwise noted, reagents were purchased from commercial suppliers and used as received. Literature procedures were used to synthesize $[U(\eta^5\text{-}C_5{}^iPr_5)_2]$,[1] $[IU(\eta^5\text{-}C_5{}^iPr_5)_2]$[2] and $KC_8$.[3] Azobenzene was purified by sublimation, and 2.2.2-cryptand and copper(I) iodide were dried under vacuum at 60°C for three days prior to use.

Elemental analyses were conducted at Mikroanalytiches Labor Pascher, Germany; [K(2.2.2-crypt)][**1**] was shipped in dry ice at –78°C. NMR spectra were recorded on a Varian VNMR S400 spectrometer operating at 400 MHz ($^1H$ frequency) and at 298 K unless otherwise stated. Chemical shifts were referenced internally to residual protons. IR spectra were recorded in a glove box using a Bruker Alpha FTIR spectrometer fitted with a Platinum ATR module. UV/Vis/NIR spectra were recorded on a Shimadzu UV-3600 Plus spectrophotometer using J-Young adapted quartz cuvettes. The baseline was corrected by a blank sample of solvent in a quartz cuvette and extinction coefficients were determined from single experiments.

Magnetic properties were measured on a QD Physical Property Measurement System in flame-sealed 7 mm NMR tubes. The DC susceptibility was only measured up to 200 K owing to thermal sensitivity of the compound. The thermal sensitivity of [K(2.2.2-crypt)][**1**] also meant that the sample could not be restrained in eicosane. It was possible to restrain [K(2.2.2-crypt)][**1**] in glass wool, however a non-negligible contribution from the background was recorded. At 2 K and magnetic fields below 20 kOe, the hysteresis properties of the unrestrained and restrained samples are very similar, with magnetic torque effects only becoming evident at higher fields. The noisier hysteresis data for the restrained sample is due to the presence of the glass wool sample restraint. Also, because of the background contribution, the DC and AC susceptibility for the restrained sample could not reliably be interpreted, hence the data reported correspond to the unrestrained sample in applied fields of 1 kOe and 0 kOe, respectively (Fig 3 and Figs S12-S14). Diamagnetic corrections were made with Pascal's constants.[4] X-band EPR spectra were recorded in CW mode on a Bruker EMX spectrometer equipped with a Bruker ER049X SuperX microwave bridge, a Bruker ER4122SHQE resonator, an Oxford Instruments ESR900 liquid-helium cryostat and an Oxford Instruments ITC503 temperature controller.

## Safety Consideration

Depleted uranium (primary isotope $^{238}U$) is a weak α-emitter (4.197 MeV) with a half-life of $4.47 \times 10^9$ years. Manipulations and reactions should be carried out in monitored fume hoods or in an inert glovebox in a radiation laboratory equipped with α- and β-counting equipment.

**Synthesis of [K(2.2.2-crypt)][1] from [U(η$^5$-C$_5$$^i$Pr$_5$)$_2$].** A dark green solution of [U(η$^5$-C$_5$$^i$Pr$_5$)$_2$] (20.1 mg, 0.025 mmol, 1 equiv) in hexane (3 mL) was added to a solid mixture of $KC_8$ (34.4 mg, 0.25 mmol) and 2.2.2-cryptand (9.4 mg, 0.025 mmol). The reaction mixture was stirred at room temperature for three days using a glass-coated magnetic stir bar. The reaction mixture gradually became colorless over three days. Thereafter, the colorless supernatant was decanted and the remaining solid washed with hexane (1.5 mL). Benzene (2 mL) was added to the solid, giving a brown solution and a bronze precipitate. The mixture was filtered on a 0.22 µm porosity filter frit into a vial to remove the excess $KC_8$ and graphite. The brown solution was then layered with hexane (3 mL) and stored overnight at room temperature, resulting in brown crystals of [K(2.2.2-crypt)][**1**] (16.5 mg, 55%). **$^1$H NMR** (400 MHz, $C_6D_6$, 298 K, *d*/ppm): 18.78 (br, s, 30 H, $^i$Pr CH$_3$, 30H); 13.97 (br, s, 10 H, $^i$Pr CH); 0.47 (br, s, 10 H, 2.2.2-crypt CH$_2$); –0.30 (br, s, 10 H, 2.2.2-crypt CH$_2$); –0.39 (br, s, 10 H, 2.2.2-crypt CH$_2$); –34.85 (br, s, 30 H, $^i$Pr CH$_3$, 30H). **Elemental analysis** for $C_{58}H_{106}N_2O_6KU \cdot 0.5(C_6H_6)$, found (calculated): C, 58.57 (58.91); H, 8.42 (8.83); N, 2.36 (2.25). These values are reasonable for a compound that is air- and temperature-sensitive, and they indicate partial loss of the lattice benzene upon drying the crystals in vacuo.

**Synthesis of [K(2.2.2-crypt)][1] from [IU(η$^5$-C$_5$$^i$Pr$_5$)$_2$].** The same procedure as that described above was used with [IU(η$^5$-C$_5$$^i$Pr$_5$)$_2$] (59.7 mg, 0.065 mmol) in hexane (7.5 mL), $KC_8$ (88.1 mg, 0.65 mmol) and 2.2.2-cryptand (48.9 mg, 0.13 mmol), and with stirring the reaction at room temperature for four days. The solid material was washed with hexane (1.5 mL) and benzene (3 mL) was added. Hexane (4.5 mL) was layered onto the brown solution and [K(2.2.2-crypt)][**1**] crystallized (44.8 mg, 58%).

**Attempted reduction of [IU(η$^5$-C$_5$$^i$Pr$_5$)$_2$] with KC$_8$ in the absence of 2.2.2-crypt.** A dark green solution of [IU(η$^5$-C$_5$$^i$Pr$_5$)$_2$] (9.4 mg, 0.01 mmol) in hexane (1.5 mL) was added to solid $KC_8$ (13.4 mg, 0.1 mmol). The reaction mixture was stirred at room temperature for five days with a glass-coated stirrer bar, which gave a dark green solution and a colorless precipitate. Analysis of the reaction mixture by $^1$H NMR spectroscopy revealed the formation of [U(η$^5$-C$_5$$^i$Pr$_5$)$_2$].

**Attempted reduction of [U(η$^5$-C$_5$$^i$Pr$_5$)$_2$] with KC$_8$ in the absence of 2.2.2-crypt.** A dark green solution of [U(η$^5$-C$_5$$^i$Pr$_5$)$_2$] (10.2 mg, 0.012 mmol) in hexane (1.5 mL) was added to solid $KC_8$ (16.8 mg, 0.12 mmol). The mixture was stirred at room temperature for five days with a glass-coated stirrer bar. No reaction was observed by $^1$H NMR spectroscopy.

**Stability of [K(2.2.2-crypt)][1] in benzene.** A light brown solution of [K(2.2.2-crypt)][**1**] (5.8 mg, 0.005 mmol) in $C_6D_6$ (0.5 mL) was monitored by $^1$H NMR spectroscopy at room temperature. Decomposition began after 12 hours, with full decomposition after five days (Figs S3-S5). The NMR spectra show the formation of [U(η$^5$-C$_5$$^i$Pr$_5$)$_2$] and [K(2.2.2-cryptand)][C$_5$$^i$Pr$_5$]. Over this time, the solution changed colour from brown to green and a grey precipitate of presumably formed, assumed to be metallic uranium.

**Stability of [K(2.2.2-crypt)][1] in THF.** A light brown solution of [K(2.2.2-crypt)][1] (4.8 mg, 0.005 mmol) in THF-D$_8$ (0.5 mL) was monitored by $^1$H NMR spectroscopy at room temperature. The initial light brown solution immediately turned light green. The NMR spectrum show a complex mixture of unidentifiable species (Fig. S6). Evaporating the THF-D$_8$ and adding $C_6D_6$ (0.5 mL) to the light green residue produced a $^1$H NMR without resonances for [K(2.2.2-crypt)][**1**] or [U(η$^5$-C$_5$$^i$Pr$_5$)$_2$].

**Reaction of [K(2.2.2-crypt)][1] with $CCl_4$.** Four drops of $CCl_4$ were added to a light brown solution of [K(2.2.2-crypt)][**1**] (4.5 mg, 0.004 mmol) in $C_6D_6$ (0.5 mL). The reaction mixture immediately changed color to light green. $^1H$ and $^{13}C$ NMR spectroscopy did not reveal the formation of $CHCl_3$ or $CH_2Cl_2$ (Figs S8, S9).

**Reaction of [K(2.2.2-crypt)][1] with one equivalent of copper(I) iodide.** Solid CuI (1.9 mg, 0.01 mmol) was added to a light brown solution of [K(2.2.2-crypt)][**1**] (12.6 mg, 0.01 mmol) in $C_6D_6$ (0.5 mL). The reaction was stirred overnight at room temperature, producing a green solution and a grey precipitate, presumably of copper and [K(2.2.2-crypt)]I. The $^1H$ NMR spectrum of the reaction mixture revealed the clean formation of $[U(\eta^5\text{-}C_5{}^iPr_5)_2]$ (Fig. S10).

**Reaction of [K(2.2.2-crypt)][1] with two equivalents of copper(I) iodide.** Solid CuI (4.2 mg, 0.022 mmol) was added to a light brown solution of of [K(2.2.2-crypt)][**1**] (13.4 mg, 0.011 mmol) in $C_6D_6$ (0.5 mL). The reaction mixture was stirred overnight at room temperature, producing a green solution and a grey precipitate, presumably of copper and [K(2.2.2-crypt)]I (Fig. S11). The $^1H$ NMR spectrum of the reaction mixture at room temperature is featureless, consistent with the formation of $[IU(\eta^5\text{-}C_5{}^iPr_5)_2]$. The solution was filtered and allowed to slowly evaporate, producing green crystals of $[IU(\eta^5\text{-}C_5{}^iPr_5)_2]$.

**Reaction of $[U(\eta^5\text{-}C_5{}^iPr_5)_2]$ with one equivalent of copper(I) iodide.** Solid CuI (4.2 mg, 0.022 mmol) was added to a green solution of $[U(\eta^5\text{-}C_5{}^iPr_5)_2]$ (13.4 mg, 0.011 mmol) in $C_6D_6$ (0.5 mL). The reaction was stirred overnight at room temperature, producing a green solution and a grey precipitate, presumably of copper and [K(2.2.2-crypt)]I. The $^1H$ NMR spectrum of the reaction mixture at room temperature is featureless, consistent with the formation of $[IU(\eta^5\text{-}C_5{}^iPr_5)_2]$. The solution was filtered and allowed to slowly evaporate, producing green crystals of $[IU(\eta^5\text{-}C_5{}^iPr_5)_2]$.

**Reaction of [K(2.2.2-crypt)][1] with azobenzene.** An orange solution of azobenzene (2.3 mg, 0.012 mmol) in $C_6D_6$ (0.2 mL) was added to a light brown solution of complex [K(2.2.2-crypt)][**1**] (15.3mg, 0.012 mmol) in $C_6D_6$ (0.3 mL). The reaction mixture immediately became green, and a dark brown precipitate formed. The $^1H$ NMR spectrum of the reaction mixture revealed the formation of $[U(\eta^5\text{-}C_5{}^iPr_5)_2]$ (Fig. S20). After mixing at room temperature for three days, the dark precipitate disappeared and the full consumption of $[U(\eta^5\text{-}C_5{}^iPr_5)_2]$ was observed, resulting in a complex mixture of species (Fig. S21). Alternatively, the dark brown precipitate immediately formed in the reaction can be separated by decanting the supernatant. The resulting solid was washed with hexane and dissolved in THF (0.4 mL). Slow diffusion of a 5:1 mixture of benzene/pentane into the THF solution yielded dark brown crystals of $[K(2.2.2\text{-crypt})][N_2Ph_2]\cdot 2(C_6H_6)$ (Fig. S22). **Elemental analysis** for $C_{30}H_{46}N_4O_6K$ found (calculated): C, 59.64 (60.27); H, 7.73 (7.76); N, 9.44 (9.37), corresponding to loss of lattice benzene upon drying the crystals.

**X-Ray Crystallography Details**

Single-crystal X-ray diffraction measurements on [K(2.2.2-crypt)][**1**]·$C_6H_6$ were carried out at the EPSRC National Crystallography Service at the University of Southampton on a Rigaku HyPix 6000HE diffractometer with a Rigaku FRE+ Rotating Anode (Mo-Kα) source, equipped with a UG2 goniometer and a HyPix 6000HE detector operating in $\omega$ scanning mode to fill the Ewald sphere at 100 K. The crystals were mounted on a MiTeGen MicroLoop from dried paraffin oil kept over 4 Å molecular sieves in a glove-box. Measurements on [K(2.2.2-crypt)][$N_2Ph_2$]·2($C_6H_6$) were carried out on an Agilent Gemini Ultra diffractometer with an Enhance Ultra (Cu-Kα) source, equipped with an Eos CCD area detector, operating in ω scanning mode to fill the Ewald sphere at 100 K. Control, integration and absorption correction were handled by the CrysAlisPro software. The crystal was mounted on a MiTeGen loop in dried Fomblin oil stored over 4 Å molecular sieves in a glovebox. Structures were solved in Olex2[5] with SHELXT[6] using intrinsic phasing and were refined with SHELXL[7] using least squares minimization. Anisotropic thermal parameters were used for the non-hydrogen atoms and isotropic parameters for the hydrogen atoms. Hydrogen atoms were added geometrically and refined using a riding model. Crystals of [K(2.2.2-crypt)][**1**]·$C_6H_6$ were twinned with a BASF of 0.4496. Twinning was handled using CrysAlisPro. In the structure of [K(2.2.2-crypt)][**1**]·$C_6H_6$ the [K(crypt)]$^+$, the uranium atoms, and the isopropyl groups are disordered. The disorder was handled using Olex2 and SIMU, DELU, ISOR, RIGU, and SADI restraints and the EADP constraint. Data with a $2\theta$ resolution of 50.2° ($d$ = 0.84) were discarded because of the $I/\sigma$ drop below the $3\sigma$ line. This is probably due to the observed disorder in the molecular structure of [K(2.2.2-crypt)][**1**]·$C_6H_6$.

**Table S1.** Crystal data and structure refinement for [K(2.2.2-crypt)][**1**]·$C_6H_6$ and [K(2.2.2-crypt)][$N_2Ph_2$]·2($C_6H_6$).

| | **[K(2.2.2-crypt)][1]·$C_6H_6$** | **[K(2.2.2-crypt)][$N_2Ph_2$]·2$C_6H_6$** |
|---|---|---|
| CCDC ref. code | 2170255 | 2169273 |
| Formula | $C_{64}H_{111}KN_2O_6U$ | $C_{42}H_{58}KN_4O_6$ |
| Crystal size (mm) | 0.2 × 0.07 × 0.02 | 0.6 × 0.5 × 0.48 |
| Crystal System | monoclinic | monoclinic |
| Space Group | $P2_1/c$ | $C2/c$ |
| Volume (Å$^3$) | 6549.4(4) | 4105.26(8) |
| $a$ (Å) | 21.0344(8) | 22.6622(3) |
| $b$ (Å) | 17.1160(5) | 8.94280(10) |
| $c$ (Å) | 19.4313(7) | 20.7666(2) |
| $\alpha$ (°) | 90 | 90 |
| $\beta$ (°) | 110.577(4) | 102.7250(10) |
| $\gamma$ (°) | 90 | 90 |
| $Z$ | 4 | 4 |
| Formula Weight | 1278.40 | 754.02 |
| Density (g cm$^{-3}$) | 1.297 | 1.220 |
| $\mu$ (mm$^{-1}$) | 2.589 | 1.533 |
| $F$(000) | 2666.0 | 1620.0 |
| Temperature (K) | 100.0(1) | 99.9(3) |
| Total Reflections | 20322 | 21588 |
| Unique Reflections | 20322 | 3991 |
| $R_{int}$ | - | 0.0465 |
| $R$ Indices [$I > 2\sigma(I)$] | $R_1$ = 0.0885 | $R_1$ = 0.0335 |
| | $wR_2$ = 0.1979 | $wR_2$ = 0.0861 |
| Largest Diff. Peak and Hole (e.A$^{-3}$) | 2.42 and –1.45 | 0.24 and –0.39 |
| GOF | 1.096 | 1.052 |

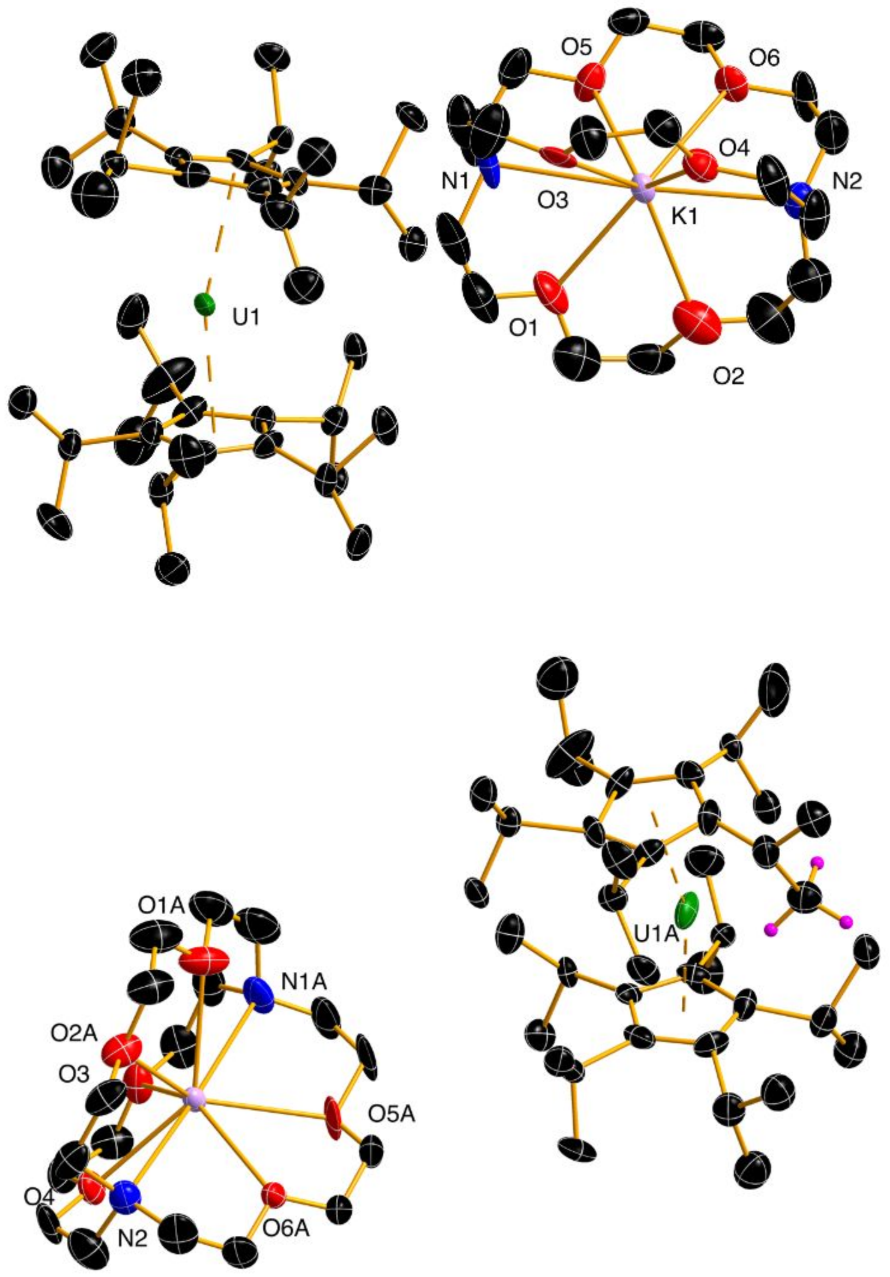


**Fig. S1.** Thermal ellipsoid representation of the structure of the two disordered forms of [K(2.2.2-crypt)][**1**] (30% probability level). The major component (68%) is shown above and the minor component (32%) below. The hydrogen atoms shown in pink are bonded to a carbon atom that resides at a relatively distance of 2.54(3) Å from the uranium centre. The other hydrogen atoms and lattice benzene have been omitted for clarity.

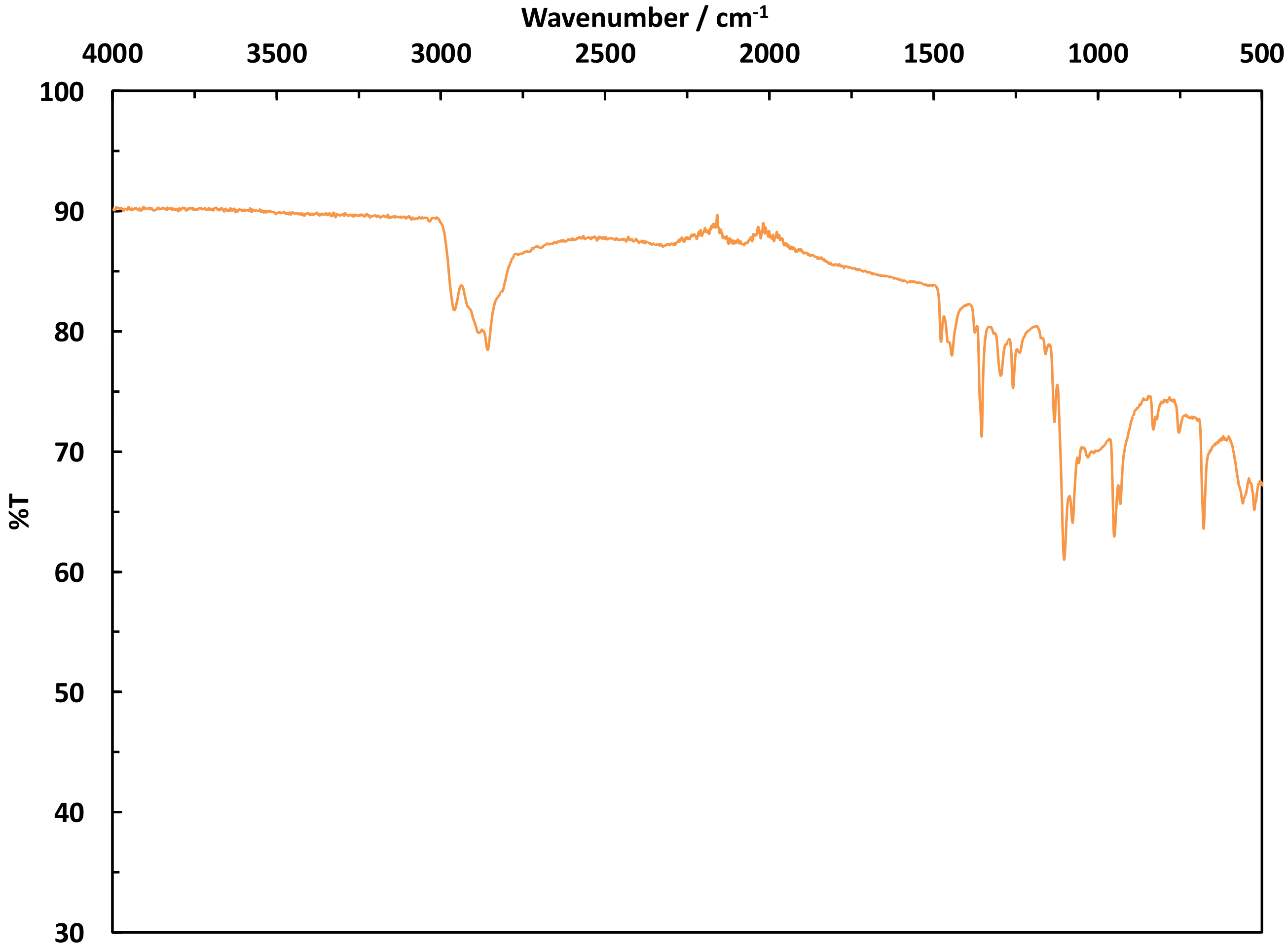


**Fig. S2.** IR spectrum of [K(2.2.2-crypt)][**1**].

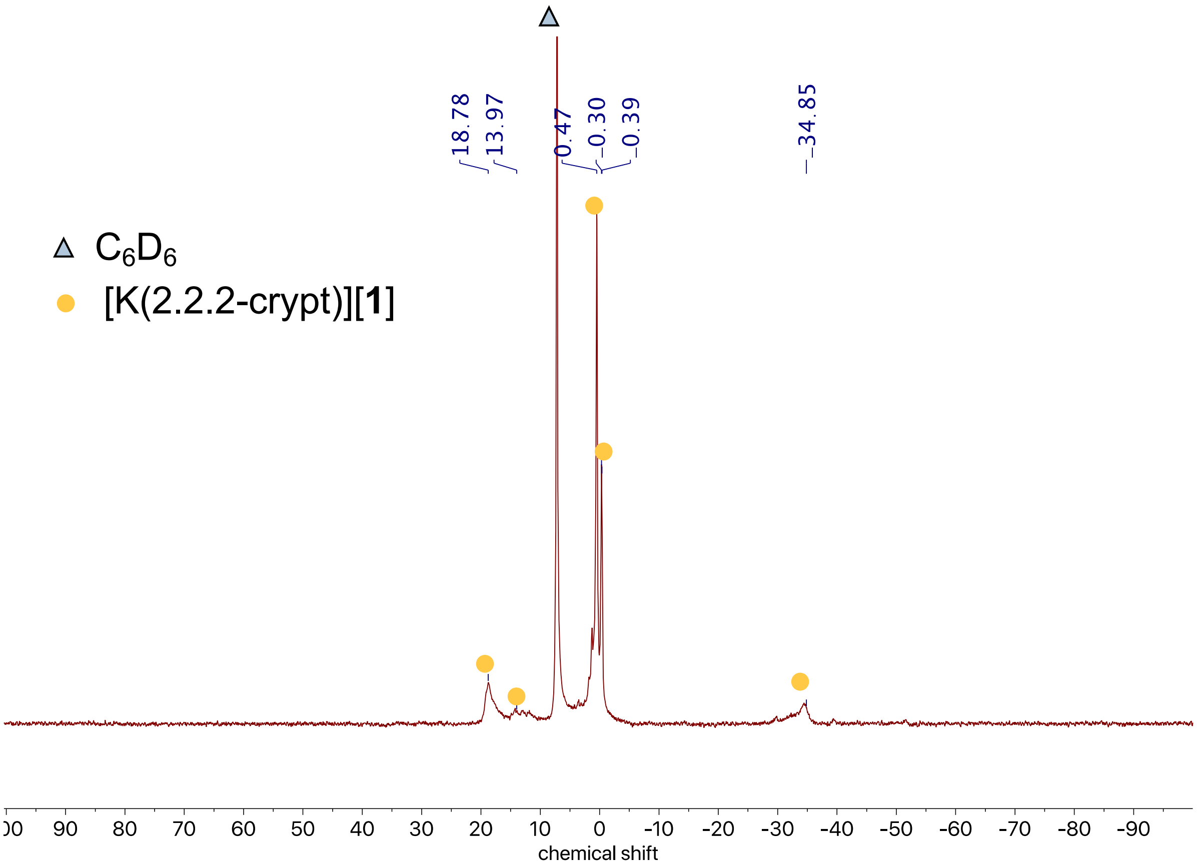


**Fig. S3.** $^1H$ NMR spectrum of [K(2.2.2-crypt)][**1**] in $C_6D_6$.

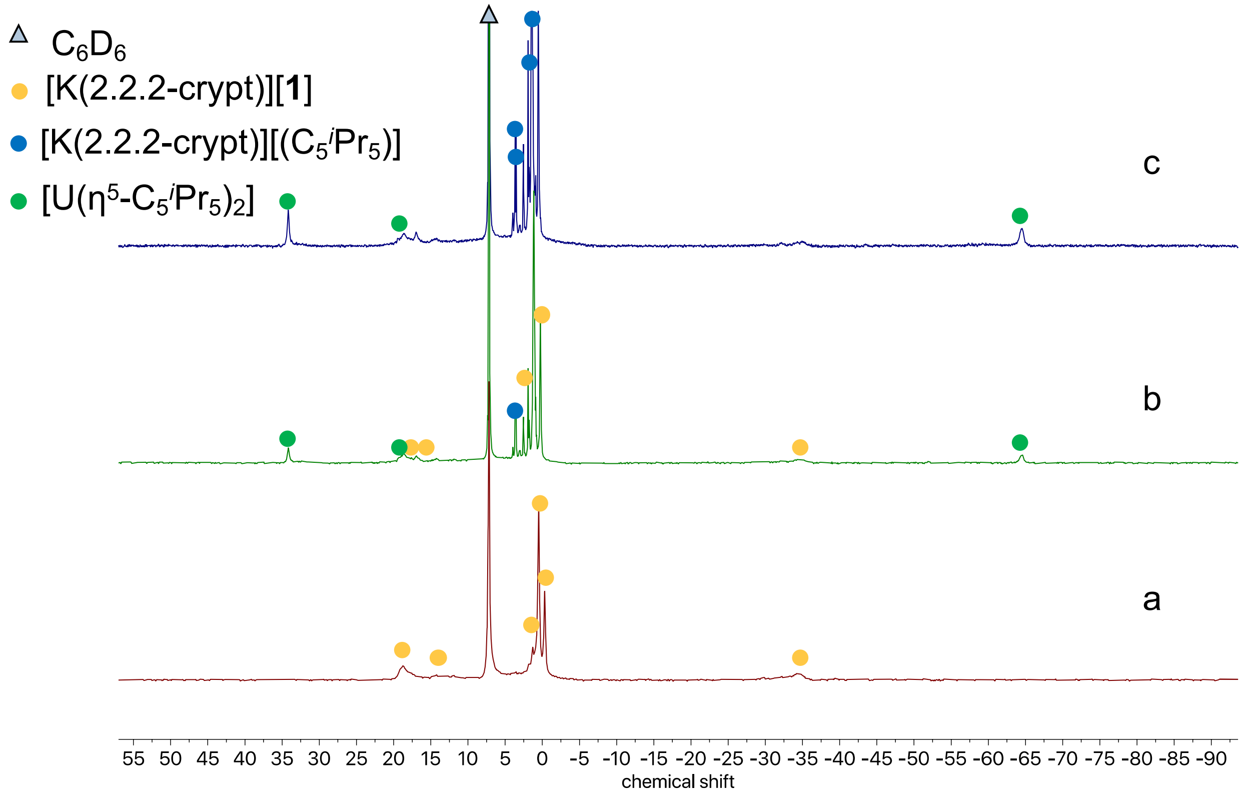


**Fig. S4.** $^1$H NMR spectra of: (a) isolated [K(2.2.2-crypt)][**1**] immediately after dissolving in $C_6D_6$; (b) after 12 hours after dissolving, and; (c) five days after dissolving.

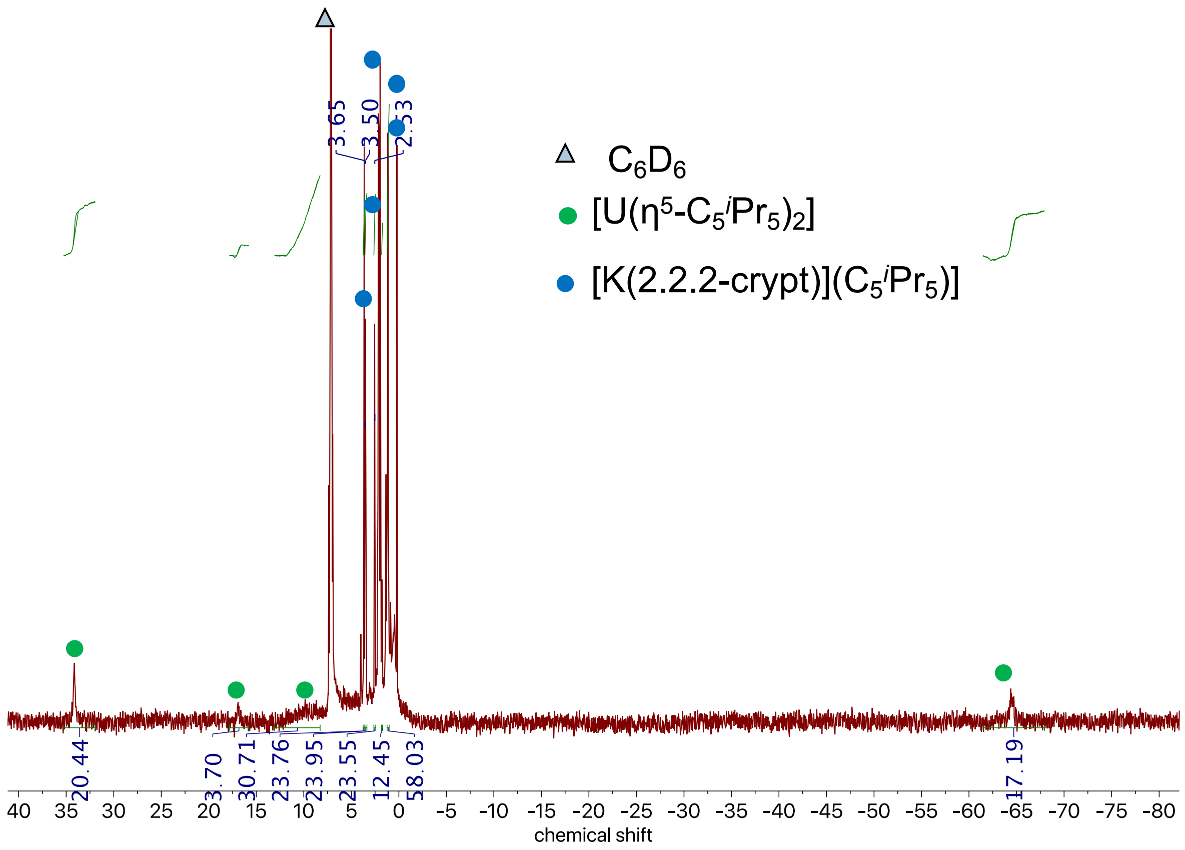


**Fig. S5.** $^{1}$H NMR spectrum of [K(2.2.2-crypt)][**1**] five days after dissolution in $C_6D_6$.

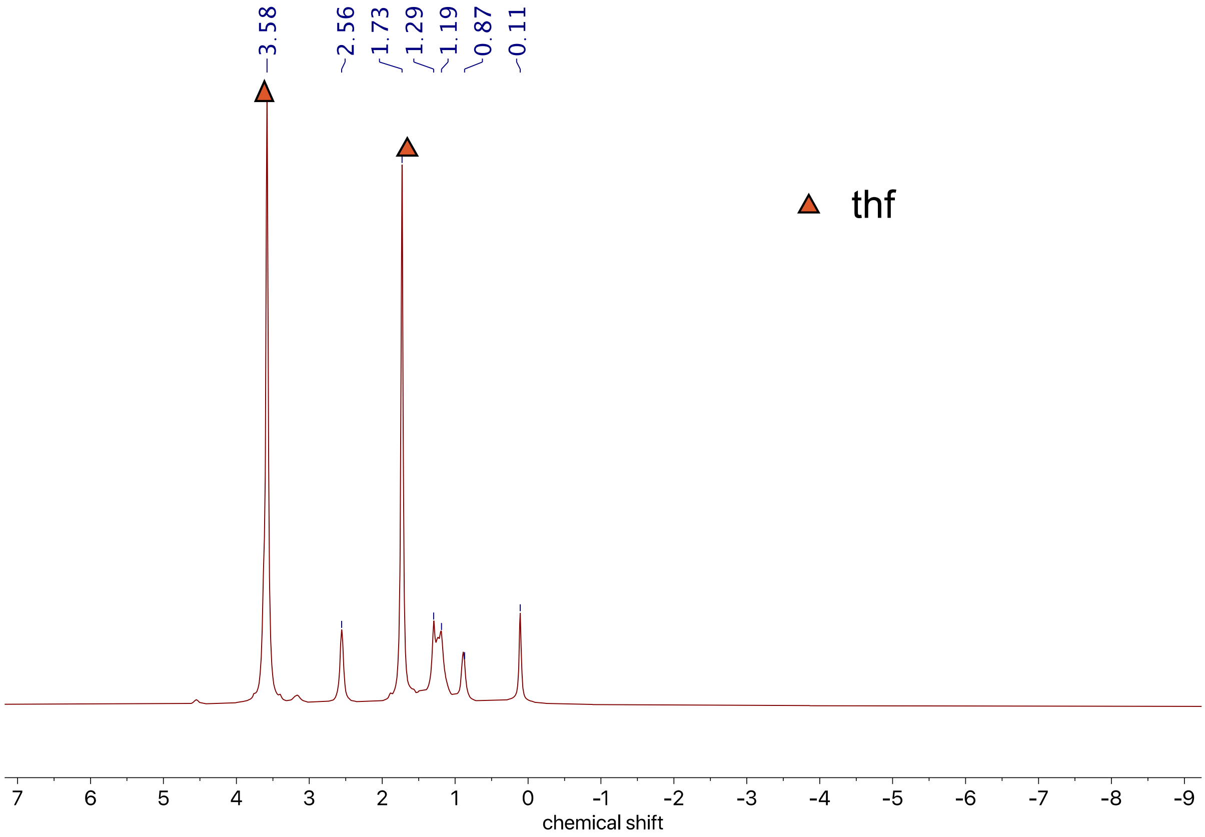


**Fig. S6.** $^{1}$H NMR spectrum of [K(2.2.2-crypt)][**1**] in THF-$D_8$.

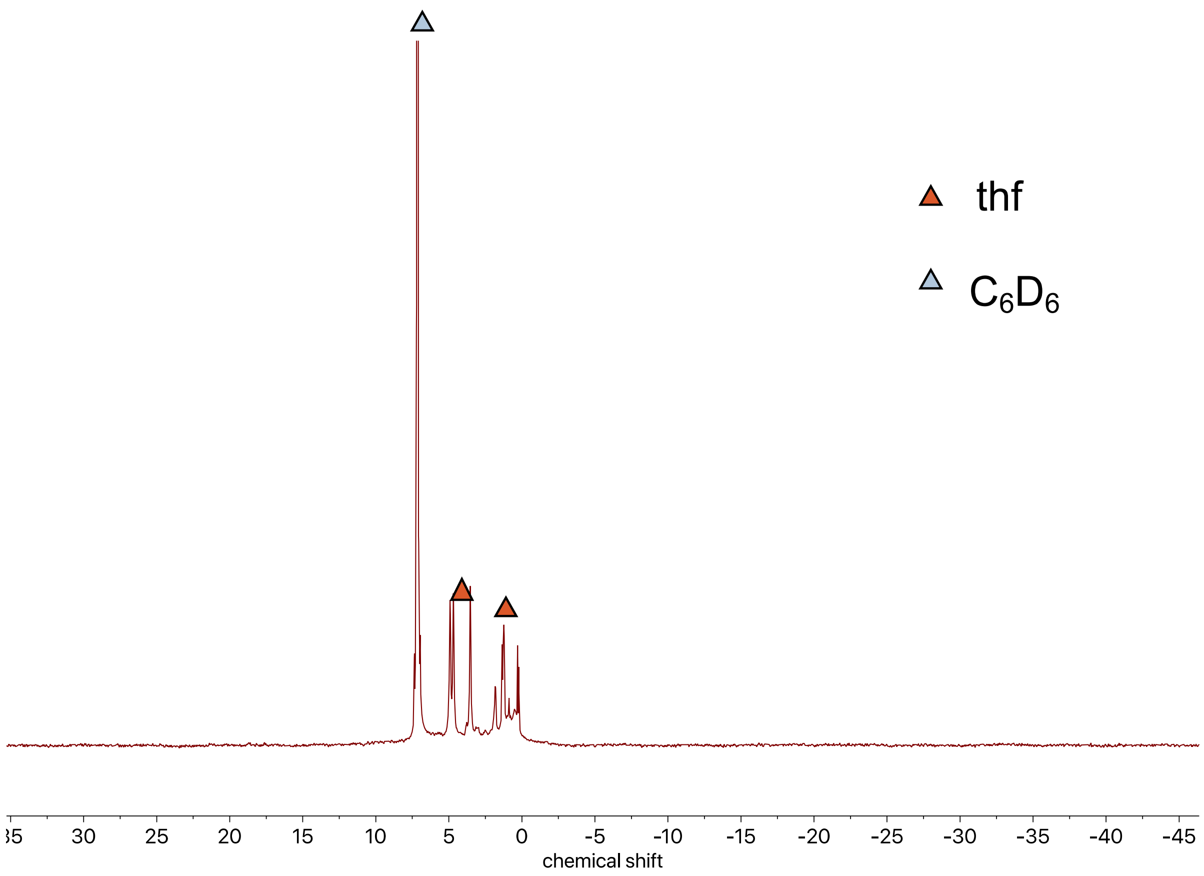


**Fig. S7.** $^1$H NMR spectrum in $C_6D_6$ of the solid residue obtained after decomposition of [K(2.2.2-crypt)][**1**] in THF-$D_8$.

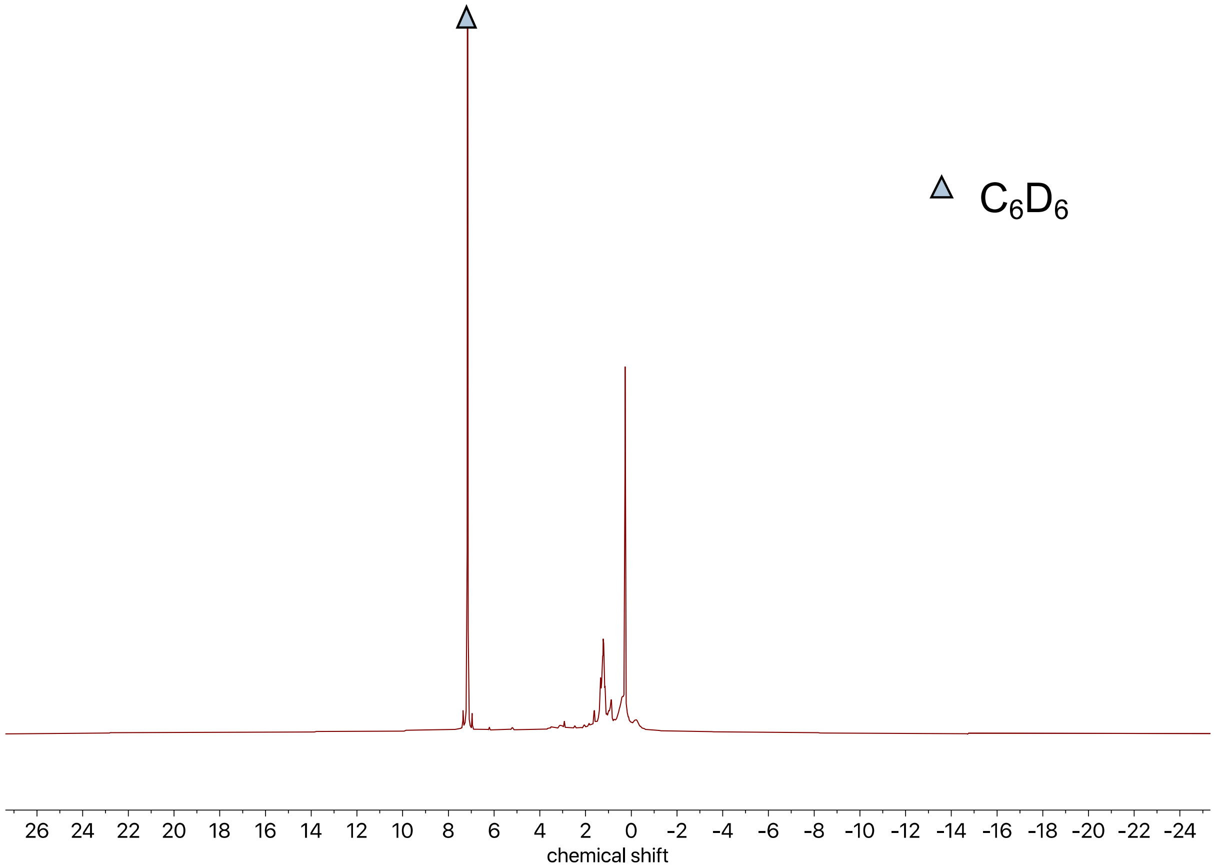


**Fig. S8.** $^1$H NMR spectrum in $C_6D_6$ after addition of an excess of $CCl_4$ to [K(2.2.2-crypt)][**1**].

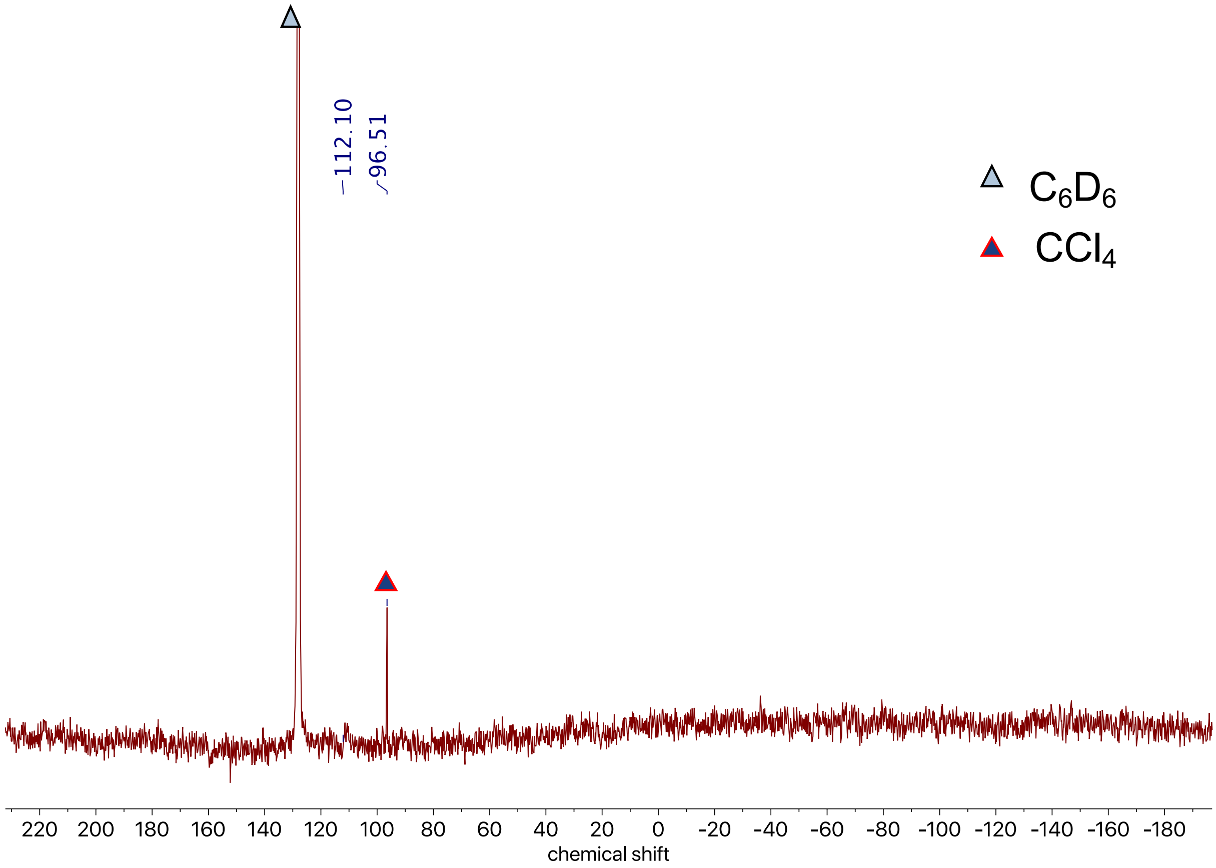


**Fig. S9.** $^{13}C$ NMR spectrum in $C_6D_6$ after addition of an excess of $CCl_4$ to [K(2.2.2-crypt)][**1**].

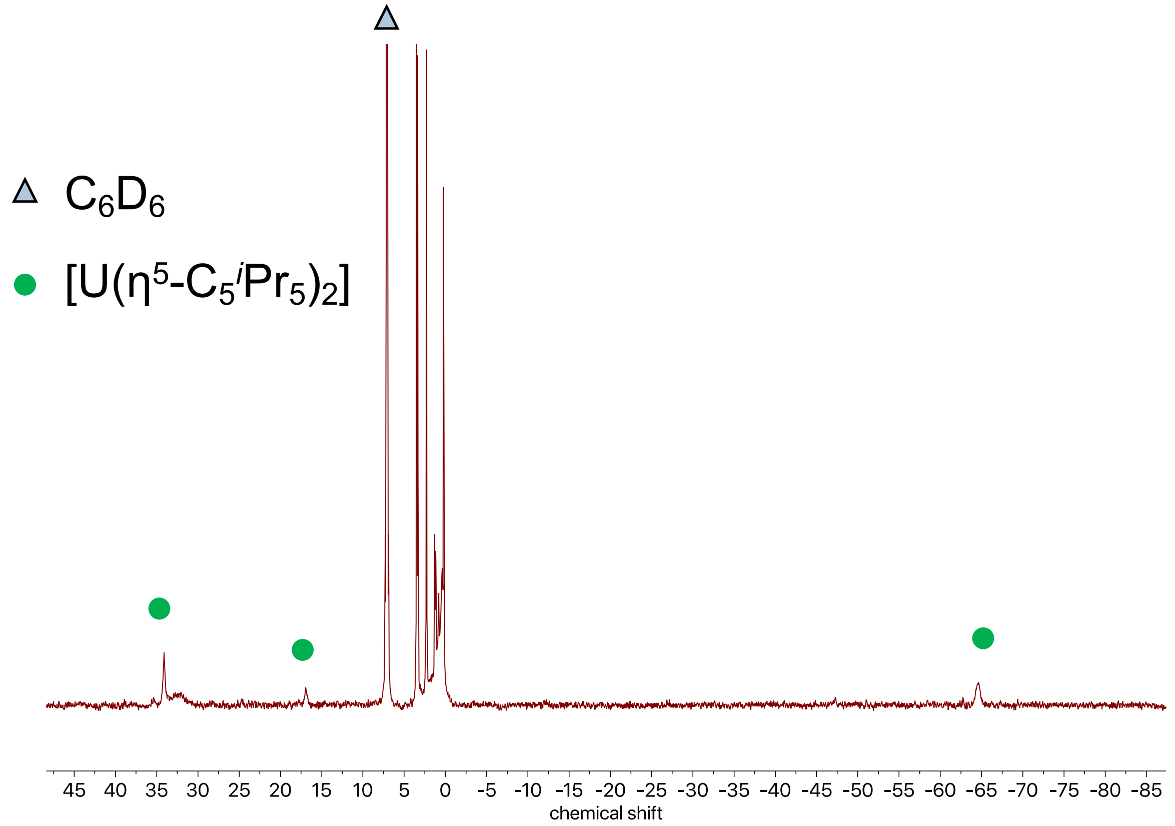


**Fig. S10.** $^1$H NMR spectrum of the reaction of [K(2.2.2-crypt)][**1**] with one equivalent of CuI.

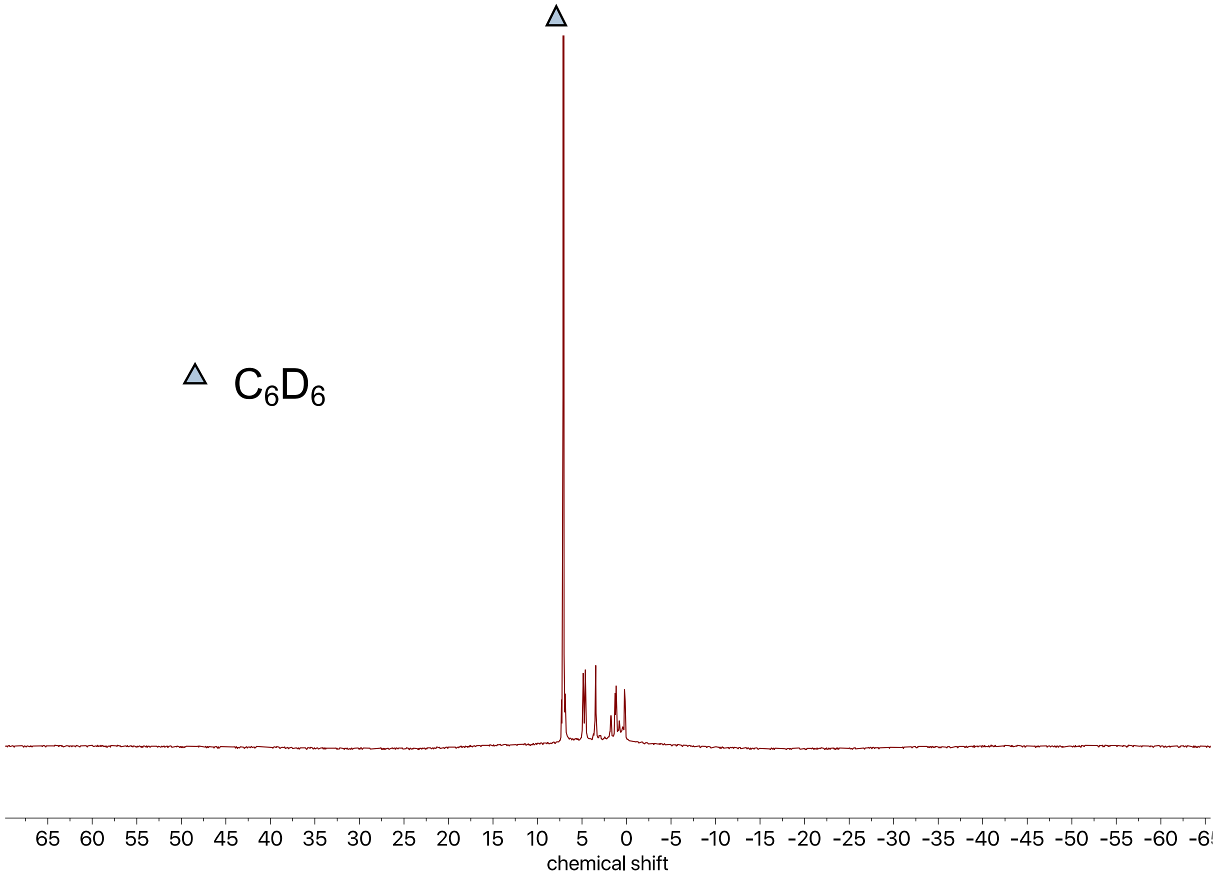


**Fig. S11.** $^{1}$H NMR spectrum of the reaction of [K(2.2.2-crypt)][**1**] with two equivalents of CuI.

**Computational details**

All calculations were carried out using density functional theory (DFT) as implemented in the ADF code[8-10] of the *Amsterdam Modelling Suite* (AMS) version 2020.101.[11] The pure PBE generalized gradient approximation[12,13] was used throughout along with the empirical DFT-D3 dispersion correction[14] utilizing the Becke-Johnson damping function.[15] Additional test calculations were carried out with the hybrid PBE0[12,13,16,17] and range-separated hybrid CAMY-B3LYP[18] exchange-correlation functionals, but no qualitative differences were observed. Scalar relativistic effects were treated with the zeroth order regular approximation (ZORA) as implemented in ADF.[19,20] Valence-triple-ζ-quality Slater-type basis sets with two sets of polarization functions (TZ2P), specifically designed for ZORA calculations, were used in all calculations for all atoms.[21,22] In geometry optimizations the 1s orbitals of carbon atoms and all orbitals up to 5d for uranium were treated as frozen core. No frozen core approximation was used in the bonding analysis. Numerical quality was set to 'Good' using the 'NumericalQuality' keyword in ADF.

Due to the strong disorder in the isopropyl groups in the crystal structure, the positions of the hydrogen atoms and the methyl groups were optimized while the positions of the remaining atoms were kept frozen to their crystal-structure coordinates. The calculations were carried out on two disordered skeleton structures extracted from the crystal structure. The major component is denoted as **1** and the results related to it are discussed in the main text. The minor component is denoted as **1'**. The electronic structures of the two geometries are very similar and only small quantitative differences are observed; thus, only the structure **1** was analyzed in detail.

The calculations were carried out with five unpaired electrons. Several test calculations were carried out to ensure that no low-lying lower-spin electronic configuration exists. For the bonding analysis, the complex was divided into metal and ligand fragments using the fragment-based approach in ADF. The U(I) ion was calculated in a restricted $5d^3 7s^1 6d^1$ configuration leading to fractional orbital occupations within the U(I) fragment. The spins were changed to an unrestricted configuration within restricted orbitals in the final calculations. The reported orbitals composition corresponds to decomposition of the molecular orbitals into contributions from the non-orthogonal fragment orbitals.

**Table S2.** Decomposition of the most important molecular orbitals in **1** into percentage contributions from non-orthogonal metal and ligand fragment orbitals.

| | 196α | 197α | 198α | 199α | 200α | 201α | 202α | 203α | 204α | 205α | 196β | 197β | 198β | 199β |
|---|---|---|---|---|---|---|---|---|---|---|---|---|---|---|
| U 7$s$ | 0.00 | 0.00 | 0.11 | 0.05 | 63.10 | 0.03 | 0.00 | 0.03 | -0.01 | -0.01 | 0.02 | 0.01 | 0.03 | 0.03 |
| U 6$d_{z2}$ | 0.84 | 3.92 | 0.27 | 0.02 | 3.19 | 0.67 | 0.01 | 31.40 | 0.17 | 11.93 | 0.41 | 2.74 | 0.17 | 0.04 |
| U 6$d_{x2-y2}$ | 0.08 | 2.89 | 1.26 | 0.00 | 12.20 | 0.30 | 0.23 | 19.59 | 0.25 | 2.13 | 0.12 | 1.88 | 0.62 | 0.00 |
| U 6$d_{xy}$ | 9.18 | 0.93 | 0.06 | 0.37 | 2.40 | 0.40 | 0.34 | 0.01 | 1.10 | 11.23 | 6.37 | 0.28 | 0.05 | 0.26 |
| U 6$d_{xz}$ | 1.76 | 5.63 | 0.60 | 0.06 | 9.83 | 0.01 | 0.31 | 10.01 | 0.01 | 0.08 | 0.80 | 3.96 | 0.42 | 0.06 |
| U 6$d_{yz}$ | 1.01 | 0.91 | 0.07 | 0.31 | 0.28 | 0.14 | 0.34 | 13.90 | 0.01 | 50.58 | 0.80 | 0.58 | 0.06 | 0.14 |
| U 5$f_{z3}$ | 0.01 | 0.00 | 0.02 | 0.22 | 0.00 | 2.10 | 0.11 | 0.26 | 18.49 | 0.00 | 0.00 | 0.00 | 0.00 | 0.00 |
| U 5$f_{z(x2-y2)}$ | 0.08 | 0.09 | 0.24 | 0.82 | 0.05 | 21.12 | 3.28 | 1.65 | 28.02 | 1.18 | 0.00 | 0.00 | 0.00 | 0.00 |
| U 5$f_{xyz}$ | 0.08 | 0.10 | 0.81 | 0.31 | 0.35 | 0.66 | 6.42 | 0.00 | 3.75 | 0.40 | 0.00 | 0.00 | 0.00 | 0.00 |
| U 5$f_{xz2}$ | 0.02 | 0.00 | 0.14 | 3.13 | 0.02 | 12.72 | 17.27 | 0.00 | 2.25 | 2.28 | 0.01 | 0.00 | 0.11 | 2.14 |
| U 5$f_{yz2}$ | 0.00 | 0.04 | 0.12 | 0.09 | 0.00 | 29.41 | 53.44 | 0.00 | 0.07 | 0.55 | 0.00 | 0.00 | 0.00 | 0.00 |
| U 5$f_{x(x2-3y2)}$ | 0.45 | 0.08 | 0.46 | 0.54 | 0.14 | 23.91 | 9.98 | 0.31 | 34.25 | 1.70 | 0.00 | 0.00 | 0.00 | 0.00 |
| U 5$f_{y(3x2-y2)}$ | 0.19 | 0.02 | 2.19 | 0.00 | 0.57 | 1.00 | 2.80 | 6.40 | 7.48 | 0.59 | 0.11 | 0.00 | 1.57 | 0.01 |
| $Cp^{iPr}$ HOMO | 49.60 | 0.82 | 34.41 | 1.62 | 0.24 | 0.01 | 0.00 | 0.01 | 0.06 | 0.00 | 49.55 | 3.19 | 37.10 | 1.29 |
| $Cp^{iPr}$ HOMO | 0.01 | 34.89 | 5.22 | 45.84 | 0.05 | 0.01 | 0.00 | 0.00 | 0.02 | 0.00 | 0.32 | 33.56 | 5.67 | 51.32 |
| $Cp^{iPr}$ HOMO | 23.82 | 20.67 | 37.33 | 5.35 | 0.21 | 0.00 | 0.00 | 0.03 | 0.09 | 0.01 | 31.45 | 19.61 | 35.30 | 5.29 |
| $Cp^{iPr}$ HOMO | 9.80 | 26.24 | 13.14 | 37.80 | 0.00 | 0.00 | 0.01 | 0.03 | 0.01 | 0.00 | 7.41 | 32.09 | 15.82 | 36.18 |

**Table S3.** Decomposition of the most important molecular orbitals in **1'** (minor disordered component) into percentage contributions from non-orthogonal metal and ligand fragment orbitals.

| | **196α** | **197α** | **198α** | **199α** | **200α** | **201α** | **202α** | **203α** | **204α** | **205α** | **196β** | **197β** | **198β** | **199β** |
|---|---|---|---|---|---|---|---|---|---|---|---|---|---|---|
| U 7*s* | 0.00 | 0.00 | 0.12 | 0.00 | 62.51 | 0.09 | 0.00 | 0.33 | 0.33 | 0.00 | 0.00 | 0.01 | 0.05 | 0.01 |
| U $6d_{z2}$ | 0.00 | 0.00 | 0.01 | 0.43 | 5.14 | 0.10 | 2.92 | 5.95 | 5.64 | 40.00 | 0.00 | 0.00 | 0.02 | 0.22 |
| U $6d_{x2-y2}$ | 0.75 | 0.23 | 0.93 | 0.12 | 20.95 | 0.16 | 0.59 | 1.30 | 0.48 | 11.47 | 0.54 | 0.15 | 0.47 | 0.11 |
| U $6d_{xy}$ | 11.49 | 1.40 | 0.14 | 0.11 | 2.14 | 0.00 | 0.71 | 0.16 | 0.23 | 0.55 | 7.87 | 0.82 | 0.05 | 0.11 |
| U $6d_{xz}$ | 2.01 | 11.89 | 0.21 | 0.03 | -0.01 | 0.23 | 0.01 | 1.27 | 0.06 | 0.70 | 1.17 | 8.11 | 0.19 | 0.02 |
| U $6d_{yz}$ | 0.01 | 0.24 | 0.10 | 0.35 | 1.36 | 0.66 | 0.06 | 25.58 | 32.78 | 15.73 | 0.00 | 0.00 | 0.00 | 0.00 |
| U $5f_{z3}$ | 0.01 | 0.00 | 0.01 | 1.95 | 0.01 | 1.82 | 14.60 | 3.63 | 2.43 | 2.88 | 0.01 | 0.00 | 0.02 | 1.32 |
| U $5f_{z(x2-y2)}$ | 0.00 | 0.00 | 0.01 | 2.80 | 0.08 | 0.22 | 8.50 | 0.09 | 0.02 | 2.47 | 0.00 | 0.00 | 0.02 | 1.88 |
| U $5f_{xyz}$ | 0.01 | 0.15 | 0.00 | 0.22 | 0.01 | 51.75 | 14.38 | 0.00 | 0.86 | 1.38 | 0.00 | 0.00 | 0.00 | 0.00 |
| U $5f_{xz2}$ | 0.00 | 0.20 | 0.00 | 0.00 | 0.01 | 0.31 | 22.80 | 17.67 | 25.01 | 3.04 | 0.00 | 0.00 | 0.00 | 0.00 |
| U $5f_{yz2}$ | 0.00 | 0.00 | 0.29 | 0.00 | 0.18 | 29.19 | 6.96 | 4.35 | 0.16 | 0.02 | 0.00 | 0.00 | 0.00 | 0.00 |
| U $5f_{x(x2-3y2)}$ | 0.29 | 0.30 | 0.49 | 0.00 | 0.09 | 2.38 | 13.92 | 26.55 | 19.14 | 3.66 | 0.00 | 0.00 | 0.00 | 0.00 |
| U $5f_{y(3x2-y2)}$ | 0.05 | 0.00 | 3.76 | 0.00 | 0.58 | 6.35 | 7.48 | 3.66 | 1.73 | 2.66 | 0.03 | 0.01 | 2.54 | 0.01 |
| $Cp^{iPr}$ HOMO | 42.40 | 1.58 | 35.05 | 7.87 | 0.09 | 0.01 | 0.00 | 0.01 | 0.01 | 0.00 | 46.09 | 2.57 | 36.58 | 6.15 |
| $Cp^{iPr}$ HOMO | 0.21 | 32.40 | 13.59 | 40.13 | 0.17 | 0.01 | 0.00 | 0.00 | 0.08 | 0.00 | 0.29 | 33.30 | 13.25 | 44.10 |
| $Cp^{iPr}$ HOMO | 31.11 | 16.54 | 38.23 | 0.64 | 0.11 | 0.01 | 0.01 | 0.03 | 0.04 | 0.01 | 33.68 | 18.13 | 38.28 | 1.05 |
| $Cp^{iPr}$ HOMO | 8.93 | 32.12 | 3.45 | 41.73 | 0.00 | 0.00 | 0.02 | 0.04 | 0.00 | 0.00 | 8.32 | 34.26 | 5.77 | 42.62 |

**Analysis of the magnetic susceptibility data**

Various theoretical values for the magnetic susceptibility calculated for **1** are listed in table S4. The observed low-temperature susceptibility (Fig. 3A) that varies between 0.98-1.17 $cm^3$ K $mol^{-1}$ in the temperature range 10–90 K is most consistent with a total *LS* coupling model. Here, the spins of all the electrons in different shells are first coupled together to give a total spin, and then coupled to the total orbital momentum. This model assumes that the 5f-7s and 5f-6d exchange coupling are stronger than the 5f *LS* coupling. This gives a magnetic susceptibility of 0.39 $cm^3$ K $mol^{-1}$ or 1.69 $cm^3$ K $mol^{-1}$ depending on whether the 6*d* orbital momentum is expected to be quenched or not, respectively. This is in reasonable agreement with the experimentally observed $\chi_M T$ value assuming that the 6d orbital contribution is partially quenched due to the strong 6d-ligand interaction. The only other value that would show reasonable agreement with the experimental susceptibility at low temperatures is that calculated for a $5f^5$ configuration, but this strongly contradicts the DFT calculations. It is also inconsistent with the previously characterized $[U(\eta^5\text{-}C_5{}^iPr_5)_2]$ (*19*), where a $7s/6_{dz^2}$ type orbital is already occupied despite the higher oxidation state of the uranium ion. It should also be noted that the free-ion $5f^37s^2$ configuration would lead to a susceptibility that is much too small. This further supports the assignment of an electronic configuration in **1** where all five valence electrons are unpaired.

**Table S4.** Magnetic susceptibilities calculated using simple theoretical models for various possible electronic configurations of **1**.

| Configuration | $\chi_M T$ / $cm^3$ K $mol^{-1}$ | | | |
|---|---|---|---|---|
| | Uncoupled [a] | Total *LS* coupling [b] | *Jjj* coupling [c] | Spin-only |
| $5f^37s^16d^1$ | 2.39 | 0.39 | 4.11 | 4.38 |
| $5f^37s^16d^1$ [d] | 2.31 | 1.69 | 4.32 | 4.38 |
| $5f^37s^2$ | 1.64 | 1.64 | 1.64 | 1.88 |
| $5f^47s^1$ | 2.01 | 0.90 | 2.74 | 3.00 |
| $5f^46d^1$ | 2.01 | 0.90 | 2.74 | 3.00 |
| $5f^46d^1$ | 1.94 | 2.68 | 2.92 | 3.00 |
| $5f^5$ | 0.88 | 0.88 | 0.88 | 1.88 |

[a] The susceptibility is calculated as the sum of the angular momentum states arising from uncoupled 5f, 7s and 6d states.

[b] The spins of all orbitals are first coupled together and the orbital momentum is then coupled opposite to the total spin momentum.

[c] The 5f, 7s and 6d electrons are first coupled to a total angular momentum state within each shell and these are then coupled parallel to each other.

[d] The orbital contribution from the 6d orbitals is assumed to be fully quenched.

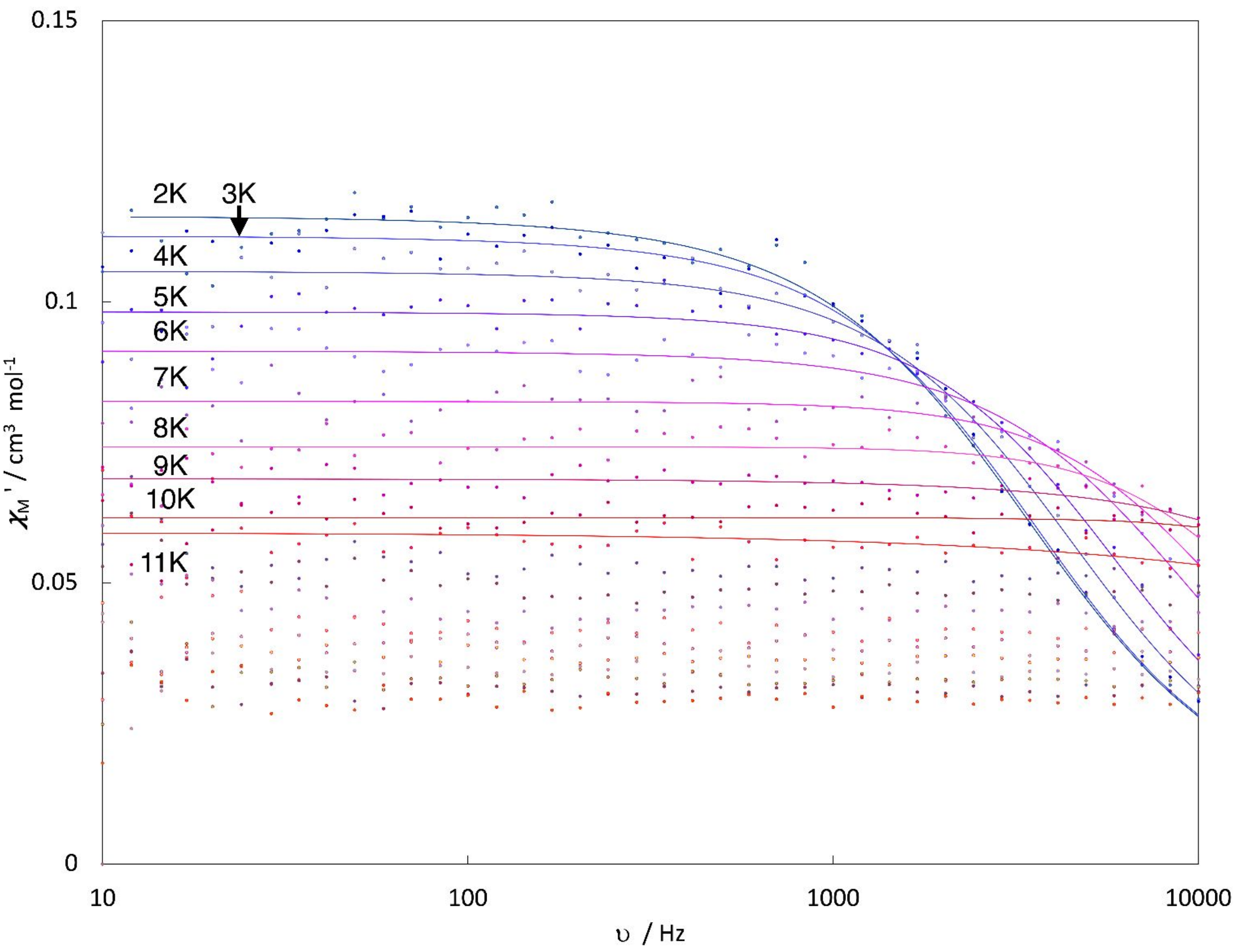


**Fig. S12.** Frequency dependence of the in-phase ($\chi'_{M}$) susceptibility for [K(2.2.2-crypt)][**1**] in zero DC field at various temperatures in the range 2.0 K (blue) to 11 K (red). Solid lines are fits to the data using equation S1:

$$\frac{\chi(\upsilon) - \chi_S}{\chi_T - \chi_S} = \frac{1}{1 + (i\upsilon\tau)^{1-\alpha}}$$

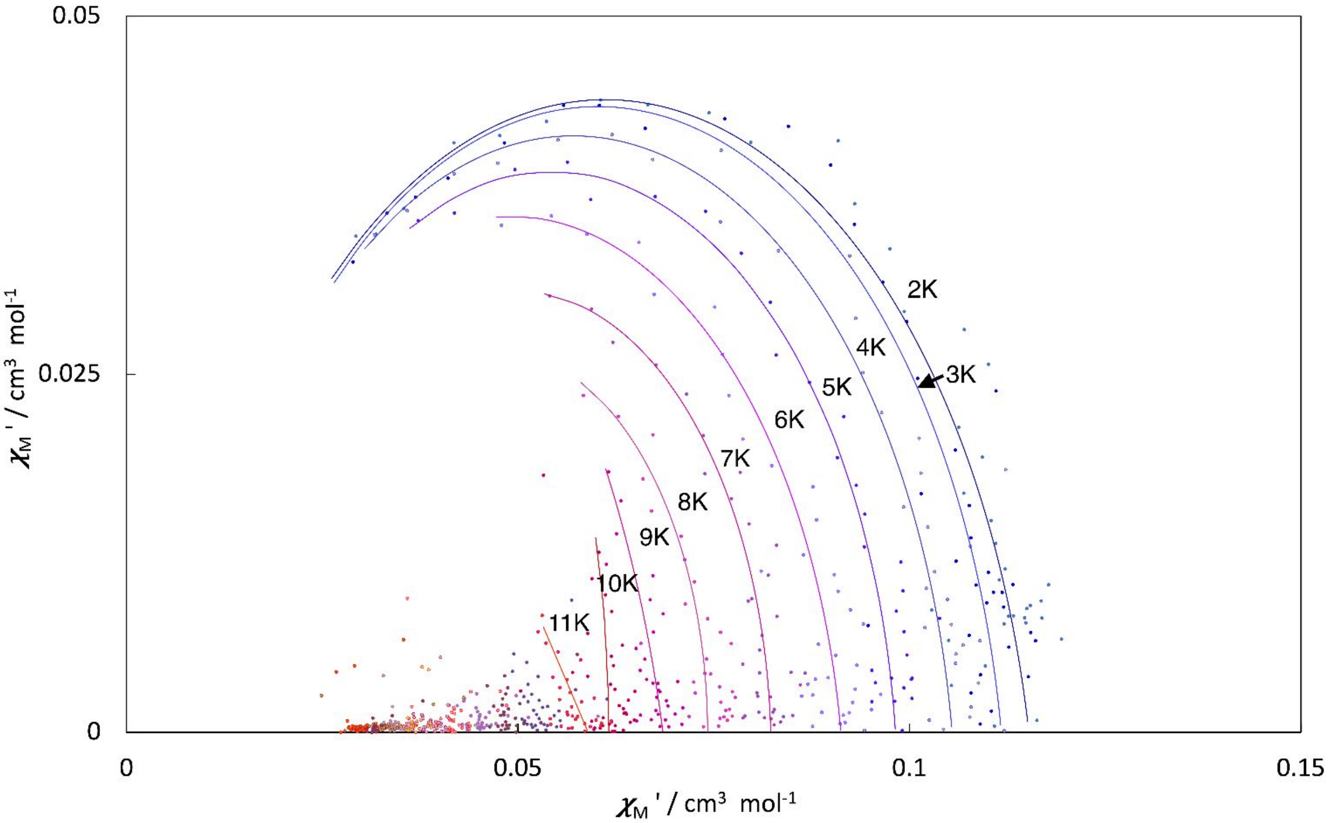


**Fig. S13.** Cole-Cole plots for the AC susceptibility in zero DC field for [K(2.2.2-crypt)][**1**] from 2-11 K. Solid lines represent fits to the data using equation S1.

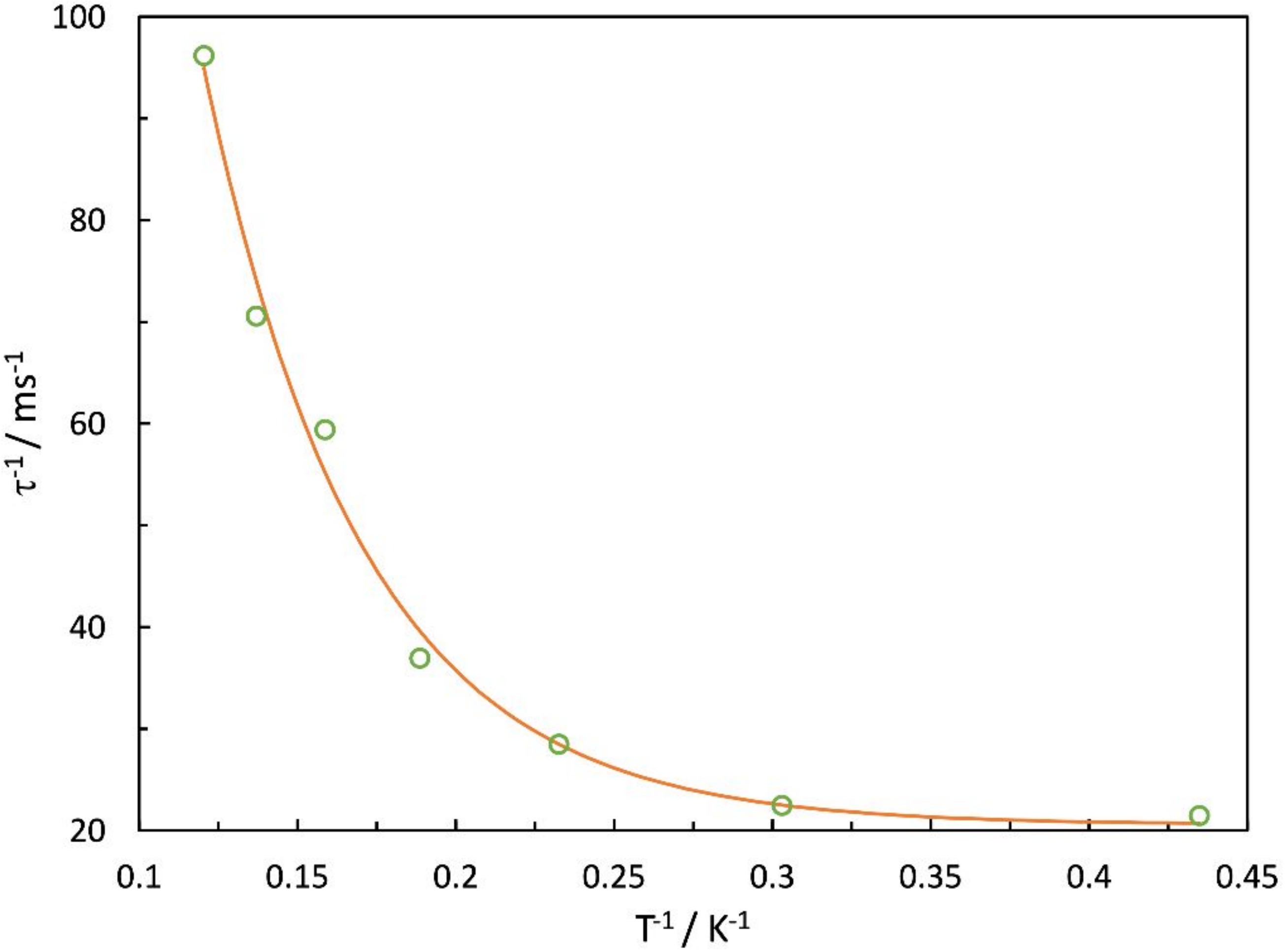


**Fig. S14.** Plot of relaxation time ($\tau$) *vs.* inverse temperature for [K(2.2.2-crypt)][**1**]. The solid red line is the best fit (adjusted $R^2$ = 0.996) to the equation $\tau^{-1} = \tau_0^{-1} e^{-U_{\mathrm{eff}}/k_{\mathrm{B}}T}$, giving $U_{\mathrm{eff}}$ = 14(1) cm$^{-1}$ and $\tau_0$ = 1.1(3) × 10$^{-7}$ s.

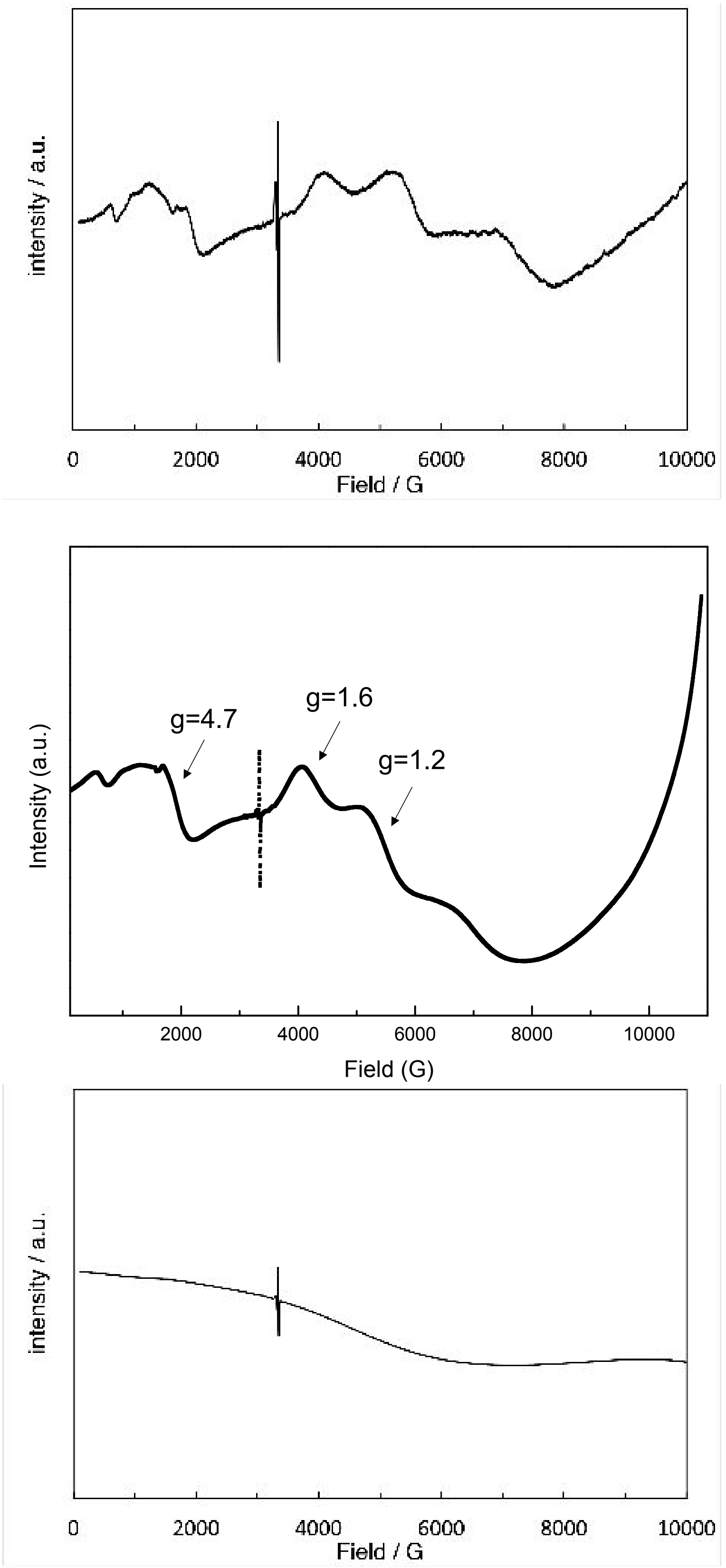


**Fig. S15.** X-Band EPR spectrum of powdered [K(2.2.2-crypt)][**1**] at 5 K (upper), 10 K (middle) and 20 K (bottom). The small narrow signal near $g = 2.0$ is attributed to an organic radical impurity.

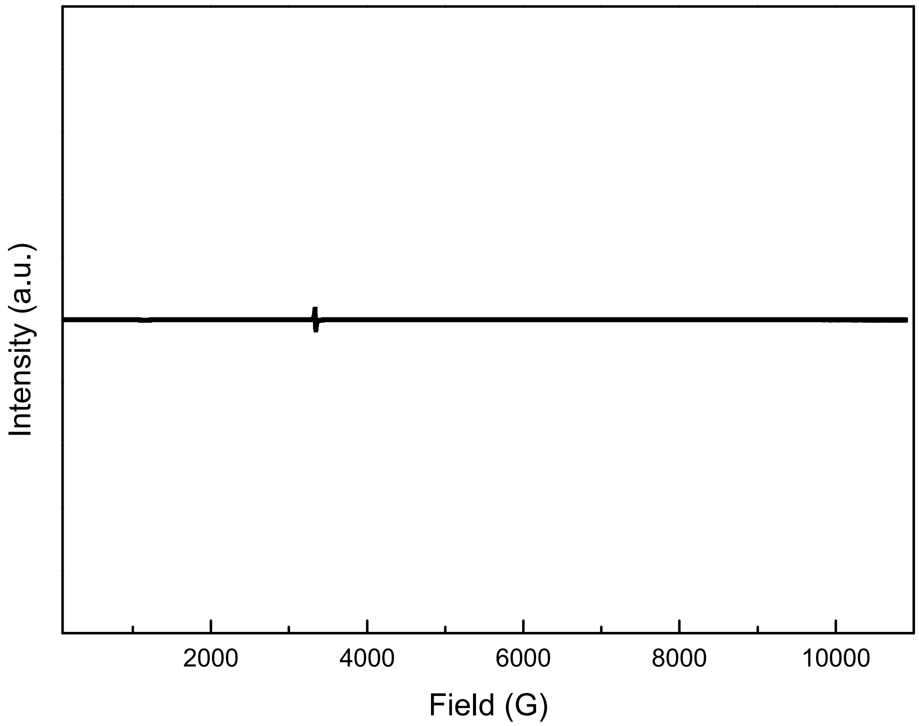


**Fig. S16.** X-Band EPR spectrum of powdered [U(η$^5$-$C_5{}^i Pr_5)_2$] at 10K. The small narrow signal near *g* = 2.0 is attributed to an organic radical impurity.

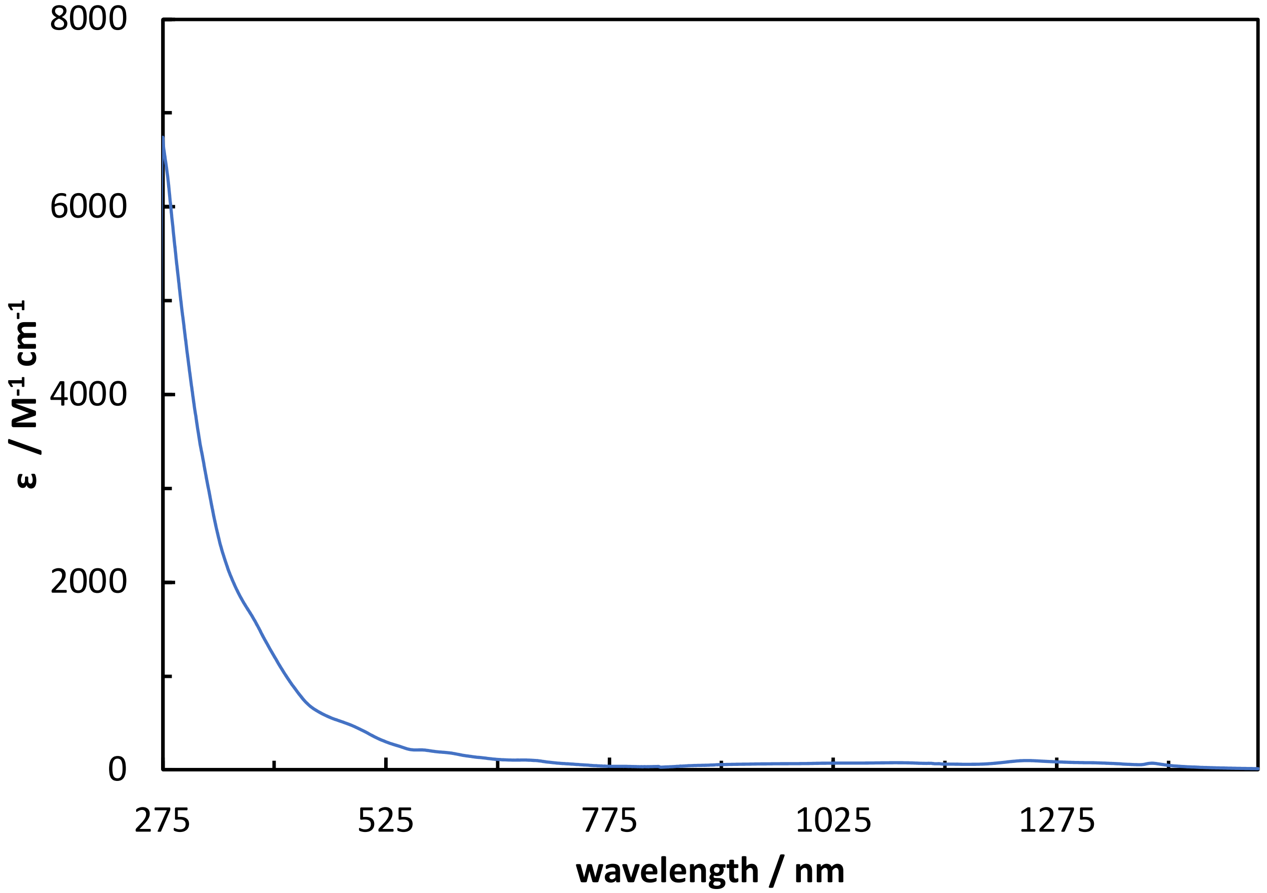


**Fig. S17.** UV/Vis/NIR spectrum of [K(2.2.2-crypt)][**1**] in benzene (0.5 mM).

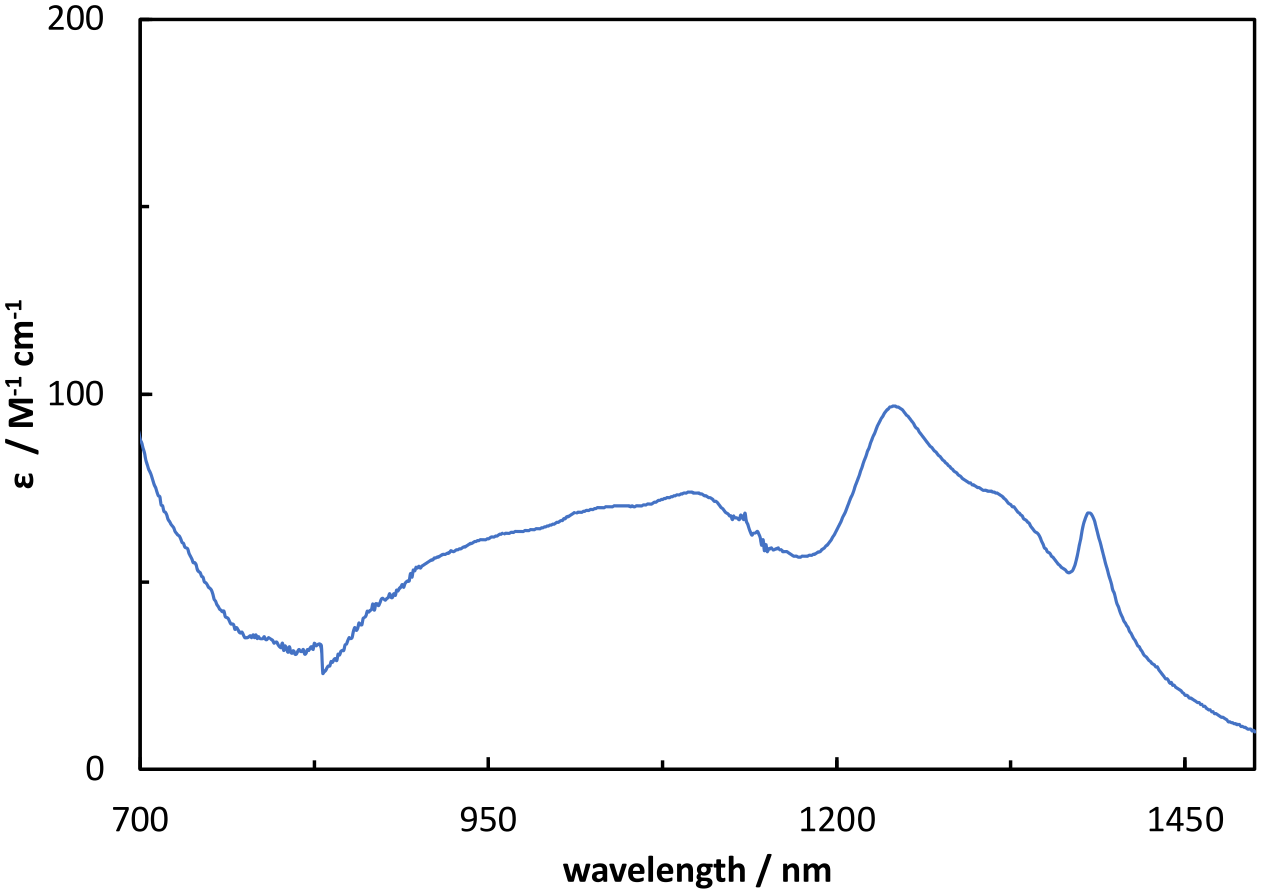


**Fig. S18.** Expanded UV/Vis/NIR spectrum of [K(2.2.2-crypt)][**1**] in benzene (0.5 mM).

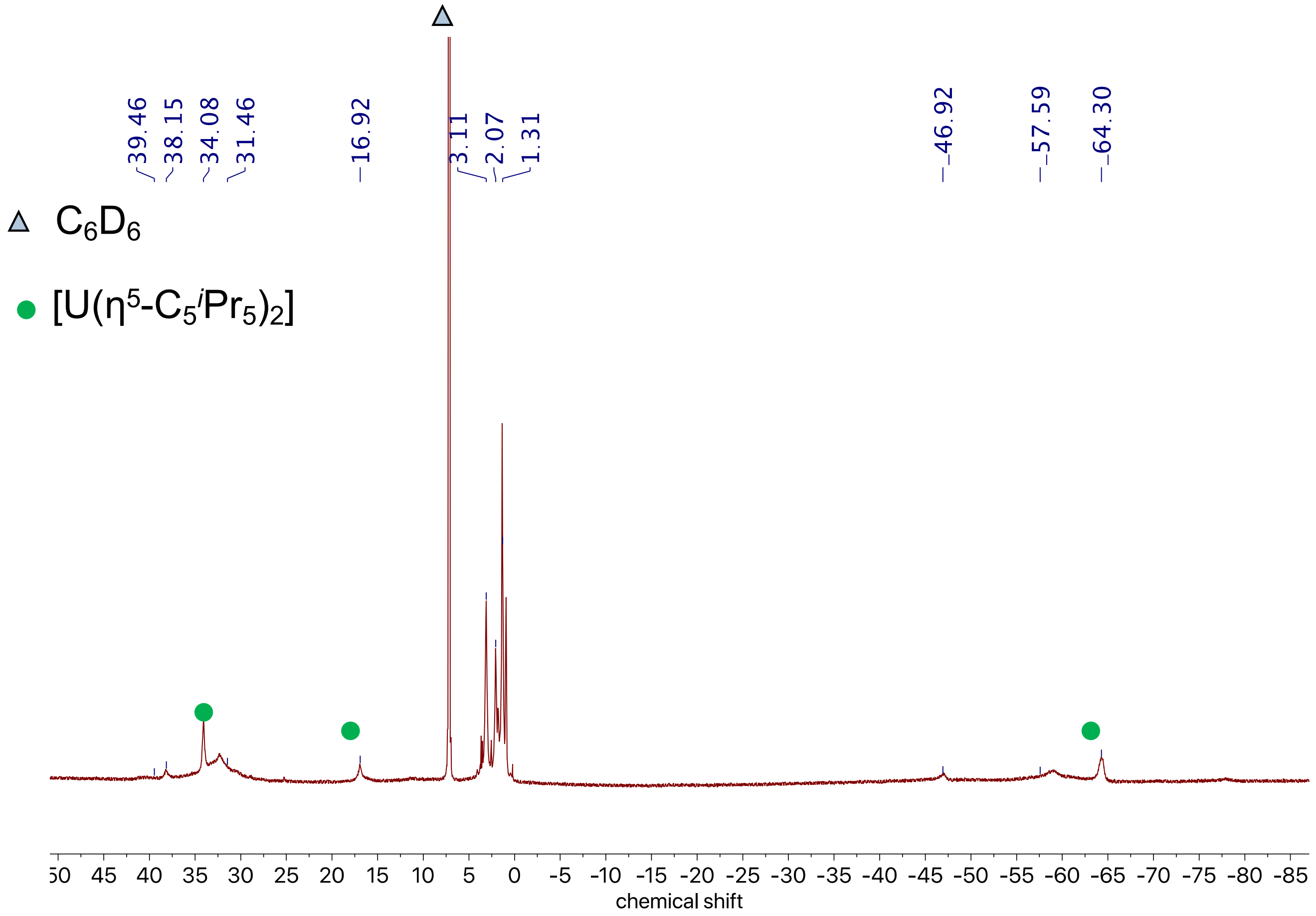


**Fig. S19.** $^{1}H$ NMR spectrum of the reaction of [K(2.2.2-crypt)][**1**] with azobenzene, recorded immediately after mixing.

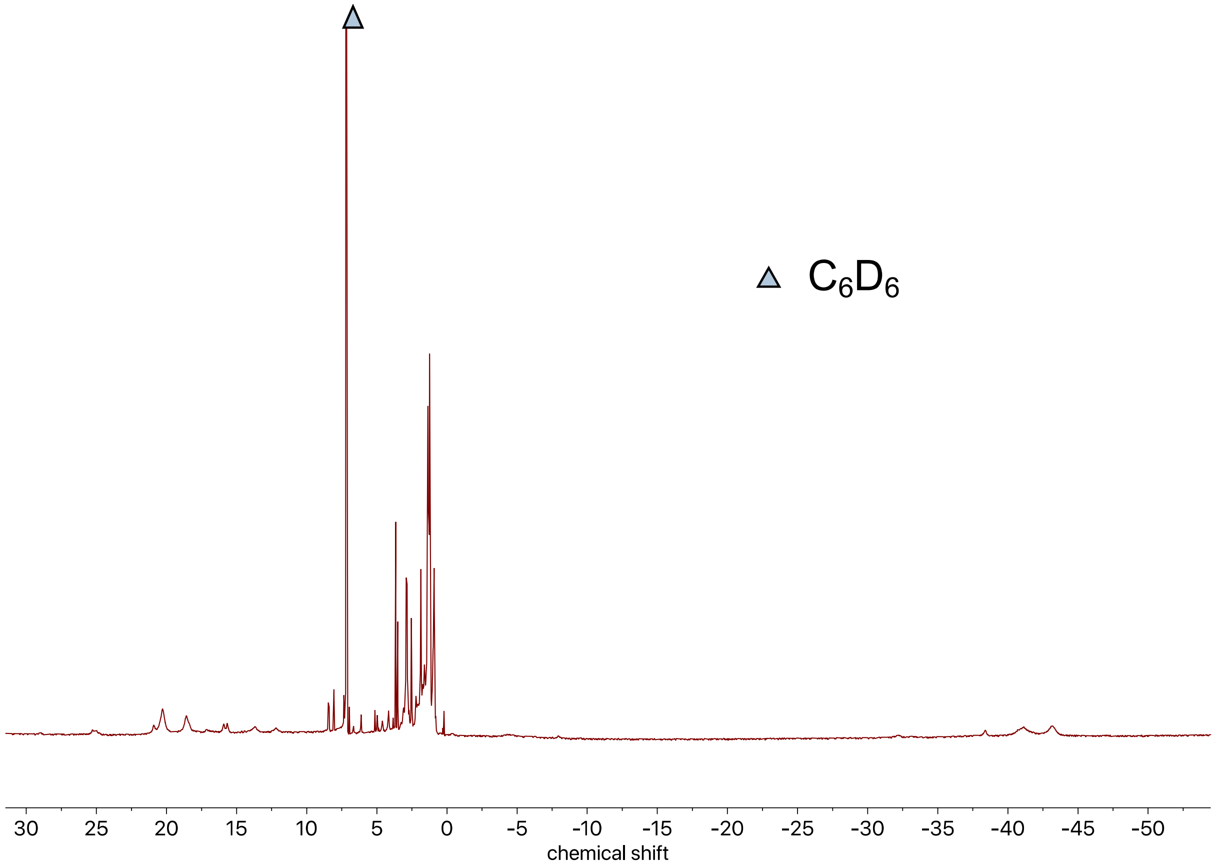


**Fig. S20.** $^{1}H$ NMR spectrum of the reaction of [K(2.2.2-crypt)][**1**] with azobenzene, recorded three days after mixing.

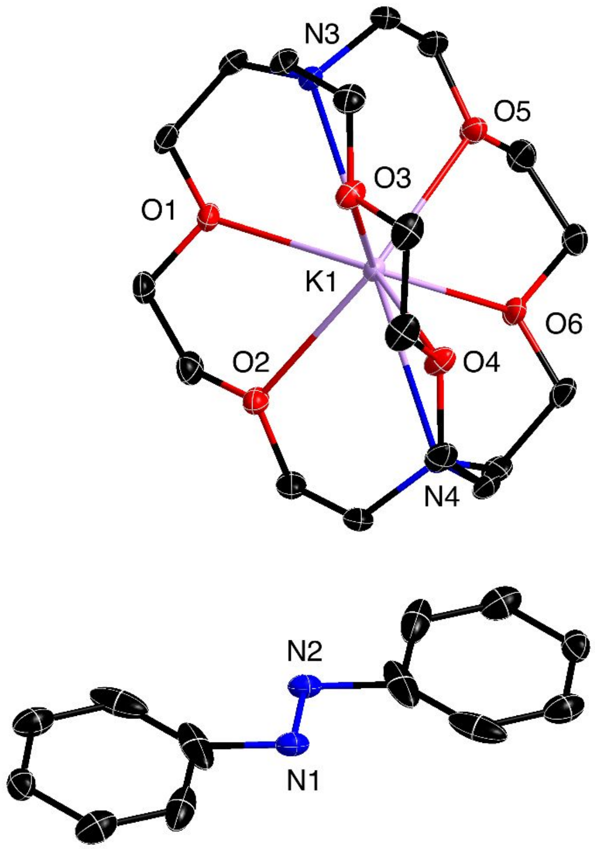


**Fig. S21.** Thermal ellipsoid representation of the structure of [K(2.2.2-crypt)][$N_2Ph_2$] (50% probability level). The hydrogen atoms and lattice benzene have been omitted for clarity. The nitrogen atoms of the azobenzene radical anion are disordered over two positions with occupancies of 65 and 35%. The average N1–N2 bond distance of 1.34(3) Å is elongated compared to azobenzene (1.25 Å).

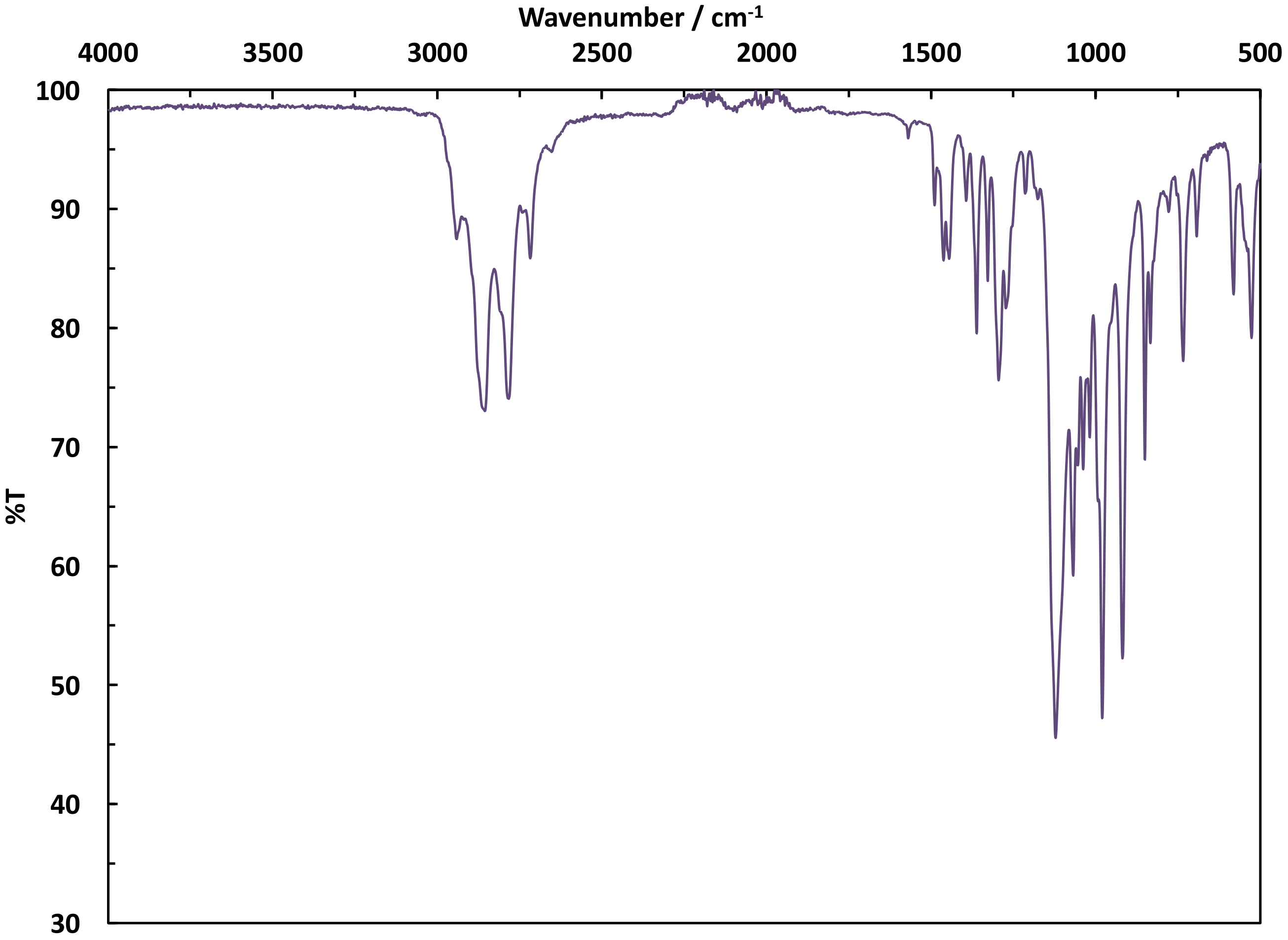

**Fig. S22.** IR spectrum of [K(2.2.2-crypt)][$N_2Ph_2$].

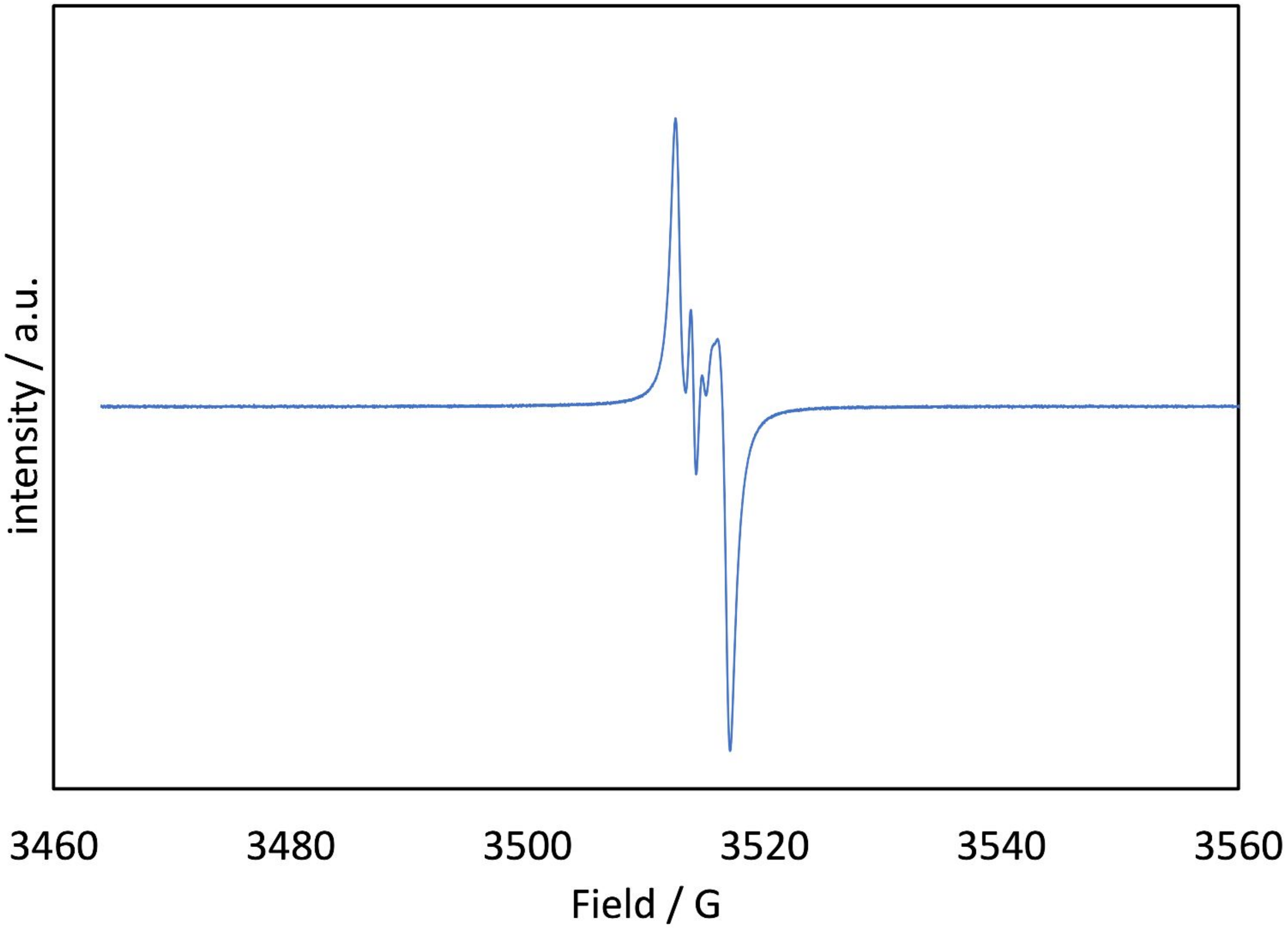


**Fig. S23.** X-Band EPR spectrum of powdered [K(2.2.2-crypt)][$N_2Ph_2$] at room temperature. The resonance is centred on $g = 2.01$.

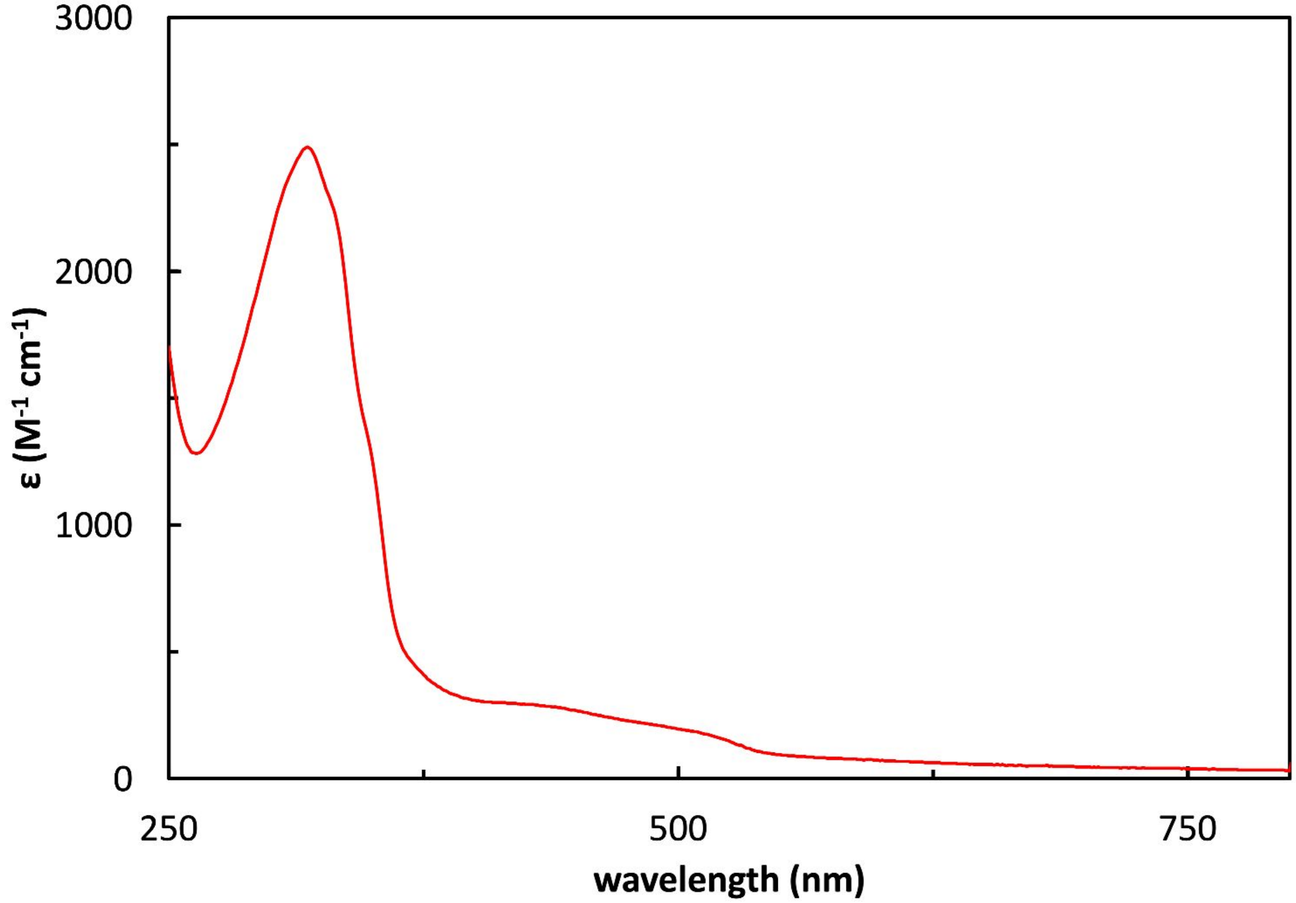

**Fig. S24.** UV/Vis/NIR spectrum of [K(2.2.2-crypt)][$N_2Ph_2$] in benzene (0.5 mM).

**Data S1**

**Optimized Cartesian coordinates for 1**

| | | | |
|---|---|---|---|
| U | 3.28854000 | 3.80261900 | 6.26629400 |
| C | 0.66672900 | 4.91105700 | 5.76037800 |
| C | 0.52956700 | 3.69087800 | 6.47419200 |
| C | 0.90192800 | 2.66316300 | 5.63898100 |
| C | 1.27677300 | 3.19222300 | 4.39881100 |
| C | 1.12620600 | 4.59532000 | 4.49560000 |
| C | 2.25518800 | 6.71631900 | 3.50367900 |
| H | 2.54656000 | 7.14069600 | 2.52947500 |
| H | 1.81051500 | 7.53034100 | 4.09245500 |
| H | 3.15975100 | 6.38897500 | 4.03043500 |
| C | 1.28906900 | 5.53730400 | 3.30936200 |
| H | 1.72829100 | 4.92985000 | 2.50775400 |
| C | -0.06448700 | 6.04243600 | 2.77574100 |
| H | -0.52585500 | 6.75309100 | 3.47481900 |
| H | 0.06547800 | 6.56129900 | 1.81219600 |
| H | -0.76808300 | 5.21184100 | 2.62997900 |
| C | 0.43539700 | 2.50118800 | 2.08852100 |
| H | 0.44697800 | 3.48475300 | 1.60049700 |
| H | 0.55521800 | 1.74012400 | 1.30065700 |
| H | -0.55333500 | 2.36957200 | 2.54902900 |
| C | 2.93276000 | 2.56738800 | 2.49619300 |
| H | 3.08283700 | 1.84082800 | 1.67927600 |
| H | 3.06013700 | 3.57369300 | 2.07338800 |
| H | 3.72836500 | 2.43375800 | 3.24466700 |
| C | 1.55154900 | 2.37354000 | 3.14416800 |
| H | 1.53409700 | 1.32514700 | 3.46724200 |
| C | 0.62442300 | 1.18576900 | 5.92724800 |
| H | 0.30558200 | 1.14000400 | 6.97521900 |
| C | 1.83052200 | 0.23557700 | 5.83727500 |
| H | 1.55661500 | -0.77385700 | 6.18841800 |
| H | 2.21708300 | 0.13606700 | 4.81310400 |
| H | 2.65891800 | 0.61258800 | 6.45911500 |
| C | -0.56185300 | 0.63832700 | 5.10787000 |
| H | -0.30800600 | 0.52626500 | 4.04547000 |
| H | -0.87168800 | -0.35187500 | 5.47942000 |
| H | -1.42380100 | 1.31670100 | 5.17204700 |
| C | -0.15735300 | 3.56081800 | 7.82956300 |
| H | -0.24731600 | 4.58171300 | 8.21810700 |
| C | 0.62142100 | 2.78404900 | 8.90670100 |
| H | 0.19617400 | 2.97510800 | 9.90531500 |
| H | 0.60186600 | 1.69751100 | 8.74512800 |
| H | 1.67604400 | 3.10064700 | 8.91621000 |
| C | -1.59726200 | 3.01956400 | 7.71689900 |
| H | -1.60834900 | 1.95750500 | 7.43715300 |
| H | -2.12789700 | 3.11490600 | 8.67816700 |
| H | -2.16417800 | 3.56877400 | 6.95275900 |
| C | 0.21973400 | 6.28866000 | 6.21715500 |
| H | 0.59388200 | 6.99256900 | 5.46343400 |
| C | -1.31510500 | 6.42893700 | 6.22139400 |
| H | -1.76348000 | 5.81370200 | 7.01323700 |
| H | -1.61139500 | 7.47491200 | 6.40263300 |
| H | -1.74689800 | 6.10657000 | 5.26462200 |
| C | 0.79317200 | 6.76861500 | 7.56287600 |
| H | 0.78475800 | 7.86797000 | 7.61794400 |
| H | 0.21530800 | 6.39674400 | 8.42007600 |
| H | 1.82810500 | 6.42723800 | 7.68820200 |
| C | 5.99112500 | 4.81695700 | 6.09984600 |
| C | 6.09298900 | 3.58279700 | 6.75233100 |
| C | 5.50478700 | 3.73532300 | 8.00286500 |
| C | 5.04625700 | 5.05251400 | 8.12628100 |
| C | 5.34179600 | 5.71978000 | 6.94471600 |
| C | 5.74469700 | 5.59043500 | 3.63519400 |
| H | 5.36904300 | 6.60676400 | 3.81278800 |
| H | 6.28149300 | 5.59947300 | 2.67220900 |
| H | 4.87426900 | 4.92249900 | 3.53938700 |
| C | 7.91281300 | 5.98637800 | 4.88589500 |
| H | 8.47605800 | 6.00163200 | 3.93879400 |
| H | 7.65290400 | 7.02440600 | 5.13223500 |
| H | 8.57801200 | 5.61355300 | 5.67715300 |
| C | 6.66305100 | 5.09366000 | 4.75975600 |
| H | 7.03038200 | 4.11955800 | 4.41306100 |

| | | | |
|---|---|---|---|
| C | 6.80924900 | 2.32491500 | 6.28126700 |
| H | 6.66345000 | 1.58539000 | 7.07912500 |
| C | 6.22338300 | 1.66695300 | 5.02105400 |
| H | 6.72786900 | 0.70818100 | 4.81392300 |
| H | 5.14846600 | 1.46498800 | 5.16517000 |
| H | 6.31791200 | 2.29886400 | 4.12806400 |
| C | 8.33340300 | 2.50955500 | 6.15738800 |
| H | 8.82912400 | 1.54014500 | 5.98893200 |
| H | 8.60349800 | 3.16891000 | 5.32212600 |
| H | 8.74358700 | 2.95286100 | 7.07546700 |
| C | 5.53660000 | 2.73825100 | 9.15831900 |
| H | 5.03008200 | 3.23515200 | 9.99494100 |
| C | 4.75789000 | 1.43096400 | 8.93935400 |
| H | 5.18708000 | 0.81315200 | 8.13884500 |
| H | 4.74621300 | 0.82645400 | 9.86270300 |
| H | 3.71664600 | 1.64506000 | 8.65084000 |
| C | 6.96824900 | 2.45000200 | 9.65016300 |
| H | 6.95021800 | 1.88517900 | 10.59612900 |
| H | 7.54058900 | 1.85794500 | 8.92346600 |
| H | 7.51592500 | 3.38772800 | 9.81729000 |
| C | 4.59900400 | 5.75652700 | 9.40406600 |
| H | 4.35770800 | 6.78481700 | 9.10325700 |
| C | 5.74186600 | 5.87380600 | 10.43273200 |
| H | 5.46459800 | 6.55969400 | 11.24947300 |
| H | 5.97850400 | 4.90107200 | 10.88470500 |
| H | 6.65817200 | 6.25087100 | 9.95928200 |
| C | 3.32446400 | 5.22762000 | 10.07947500 |
| H | 3.45901800 | 4.22495500 | 10.50794500 |
| H | 3.01715800 | 5.89885500 | 10.89895600 |
| H | 2.50513700 | 5.15916600 | 9.35214900 |
| C | 5.32103800 | 7.21965200 | 6.67190900 |
| H | 5.63714700 | 7.32929200 | 5.62652900 |
| C | 6.36892500 | 7.97575100 | 7.51258000 |
| H | 6.48880900 | 9.00917300 | 7.14893500 |
| H | 6.07334900 | 8.02957500 | 8.56906200 |
| H | 7.34570600 | 7.47581200 | 7.46416600 |
| C | 3.96300300 | 7.92594100 | 6.75341800 |
| H | 3.58375700 | 7.99199200 | 7.78182000 |
| H | 4.04282900 | 8.95497400 | 6.36615800 |
| H | 3.21854500 | 7.38275200 | 6.15826200 |

1’

| | | | |
|---|---|---|---|
| U | 0.08695700 | -0.13624600 | -0.10705200 |
| C | -2.54234400 | 0.97257000 | 0.43644200 |
| C | -2.45209700 | -0.17077200 | 1.27417900 |
| C | -2.41732000 | -1.28713200 | 0.47108400 |
| C | -2.48385100 | -0.89070800 | -0.86938400 |
| C | -2.55811800 | 0.52180900 | -0.87009500 |
| C | -3.75208000 | -0.58342700 | 3.45748900 |
| H | -3.82621800 | -1.67853000 | 3.43908900 |
| H | -3.78848800 | -0.26761800 | 4.51228700 |
| H | -4.63615000 | -0.17708400 | 2.94555600 |
| C | -3.97140800 | -3.20185800 | 1.19450800 |
| H | -4.35196100 | -3.56599000 | 0.23383300 |
| H | -4.02751700 | -4.03788800 | 1.91108700 |
| H | -4.64703400 | -2.40914600 | 1.53834800 |
| C | -1.61064900 | -3.77005000 | 0.50276100 |
| H | -1.57404800 | -4.63836000 | 1.18003800 |
| H | -1.96456200 | -4.13124800 | -0.47107200 |
| H | -0.58508500 | -3.38488600 | 0.38170200 |
| C | -2.52083300 | -2.68359900 | 1.08542800 |
| H | -2.18037200 | -2.55517000 | 2.11982100 |
| C | -1.46643100 | -2.43890400 | -2.72044500 |
| H | -1.13415200 | -3.25322700 | -2.06598400 |
| H | -1.70324500 | -2.87341100 | -3.70805300 |
| H | -0.61192700 | -1.75538400 | -2.83562600 |
| C | -3.91825100 | -2.61533300 | -2.16814500 |
| H | -4.78580000 | -2.12575200 | -1.70473900 |
| H | -4.18177000 | -2.88376500 | -3.20340400 |
| H | -3.73007300 | -3.55383800 | -1.63180100 |
| C | -2.68831100 | -1.69217700 | -2.15759800 |
| H | -2.90999800 | -0.92997400 | -2.91192800 |
| C | -2.88624100 | 1.42019800 | -2.05899000 |

| | | | |
|---|---|---|---|
| H | -2.84948400 | 2.44522400 | -1.67005000 |
| C | -4.33143300 | 1.22708100 | -2.56251500 |
| H | -4.59749300 | 2.00960000 | -3.29142000 |
| H | -4.47509000 | 0.25499700 | -3.05325700 |
| H | -5.03998800 | 1.27858600 | -1.72468600 |
| C | -1.88217700 | 1.40356300 | -3.22239200 |
| H | -1.86552100 | 0.44804000 | -3.76326000 |
| H | -2.12185400 | 2.19415600 | -3.95237200 |
| H | -0.86589800 | 1.58294800 | -2.84088600 |
| C | -1.75441500 | 3.44488700 | 0.43399400 |
| H | -1.90920400 | 3.70532200 | -0.62100000 |
| H | -1.85000200 | 4.37338800 | 1.02014500 |
| H | -0.72525500 | 3.07866200 | 0.53505200 |
| C | -2.74301700 | 2.38933300 | 0.94707200 |
| H | -2.56218500 | 2.35116600 | 2.03169500 |
| C | -4.19876500 | 2.86426000 | 0.78543700 |
| H | -4.35663700 | 3.82489500 | 1.30164300 |
| H | -4.46252200 | 3.00643300 | -0.27174300 |
| H | -4.89529100 | 2.12554900 | 1.20635500 |
| C | -2.45565200 | -0.08255400 | 2.79567100 |
| H | -2.41520300 | 0.99322200 | 3.02100500 |
| C | -1.22019100 | -0.65711300 | 3.50103100 |
| H | -1.20178700 | -0.34752400 | 4.55915700 |
| H | -1.17385100 | -1.75378600 | 3.47697300 |
| H | -0.30827900 | -0.27487500 | 3.01863600 |
| C | 2.56496500 | 0.60258400 | -1.06415900 |
| C | 2.85768100 | -0.57375100 | -0.36429000 |
| C | 2.74395700 | -0.27915200 | 0.98972600 |
| C | 2.38817400 | 1.06795200 | 1.12830100 |
| C | 2.27091600 | 1.61149600 | -0.14429200 |
| C | 1.02711500 | 2.53805600 | 2.79164100 |
| H | 0.98830400 | 2.80309600 | 3.86113600 |
| H | 0.93405100 | 3.47280700 | 2.22146900 |
| H | 0.15504300 | 1.91285100 | 2.54667000 |
| C | 2.32948600 | 1.78590300 | 2.47260500 |
| H | 2.39829600 | 0.99283100 | 3.22893600 |
| C | 3.53887900 | 2.71160600 | 2.71157000 |
| H | 3.47947900 | 3.61360600 | 2.08774700 |
| H | 3.57594300 | 3.03939300 | 3.76292900 |
| H | 4.48274100 | 2.20219000 | 2.47491300 |
| C | 2.12730700 | -1.65382800 | 3.11445800 |
| H | 2.51428800 | -2.45358000 | 3.76773800 |
| H | 1.81859700 | -0.82085600 | 3.76023000 |
| H | 1.22942300 | -2.02467000 | 2.59813800 |
| C | 4.48705800 | -0.79378500 | 2.77858600 |
| H | 4.32386800 | 0.07792900 | 3.42580400 |
| H | 4.89091800 | -1.60253000 | 3.40873500 |
| H | 5.24988800 | -0.51988300 | 2.03642000 |
| C | 3.18793500 | -1.24504300 | 2.08427900 |
| H | 3.44205900 | -2.17597700 | 1.56280300 |
| C | 3.37222000 | -1.85675400 | -1.00094500 |
| H | 3.31319900 | -1.69647100 | -2.08504500 |
| C | 4.86123600 | -2.12151000 | -0.70214400 |
| H | 5.03248900 | -2.39711800 | 0.34671400 |
| H | 5.24102000 | -2.94549300 | -1.32732800 |
| H | 5.46215800 | -1.22554300 | -0.91023300 |
| C | 2.51637500 | -3.10576600 | -0.73545400 |
| H | 2.89667400 | -3.96685600 | -1.30984000 |
| H | 2.49611200 | -3.38914800 | 0.32532900 |
| H | 1.47433200 | -2.91783900 | -1.04178400 |
| C | 4.28345400 | 0.73624200 | -2.94516600 |
| H | 4.63215500 | -0.30496900 | -2.93026300 |
| H | 4.44906900 | 1.12569400 | -3.96259500 |
| H | 4.91492200 | 1.30603100 | -2.24962500 |
| C | 2.79699700 | 0.84963000 | -2.55230800 |
| H | 2.52267100 | 1.89590000 | -2.72774800 |
| C | 1.91863700 | 0.03857800 | -3.51819900 |
| H | 2.07349500 | 0.37563700 | -4.55714200 |
| H | 2.13095900 | -1.03840400 | -3.48092400 |
| H | 0.85448000 | 0.16500800 | -3.27067400 |
| C | 2.18465800 | 3.10790500 | -0.42888700 |
| H | 1.85179800 | 3.56917900 | 0.51040000 |
| C | 1.17433100 | 3.56419300 | -1.49893900 |
| H | 0.87086300 | 4.60776400 | -1.32290700 |

| | | | |
|---|---|---|---|
| H | 1.59199000 | 3.51854300 | -2.51464800 |
| H | 0.27630100 | 2.93186200 | -1.47853200 |
| C | 3.57186900 | 3.70644400 | -0.74037700 |
| H | 3.93259400 | 3.36304000 | -1.71987400 |
| H | 3.52873800 | 4.80753700 | -0.76803300 |
| H | 4.31299200 | 3.40613900 | 0.01137800 |

References


(1) Guo, F.-S.; Tsoureas, N.; Huang, G.-Z.; Tong, M.-L.; Mansikkamäki, A.; Layfield, R. A. Isolation of a Perfectly Linear Uranium(II) Metallocene. *Angew. Chemie Int. Ed.* **2020**, *59* (6), 2299–2303. https://doi.org/10.1002/anie.201912663.

(2) Guo, F.-S.; Chen, Y.-C.; Tong, M.-L.; Mansikkamäki, A.; Layfield, R. A. Uranocenium: Synthesis, Structure, and Chemical Bonding. *Angew. Chemie Int. Ed.* **2019**, *58* (30), 10163–10167. https://doi.org/10.1002/anie.201903681.

(3) Evans, W. J.; Kozimor, S. A.; Ziller, J. W.; Kaltsoyannis, N. Structure, Reactivity, and Density Functional Theory Analysis of the Six-Electron Reductant, [(C5Me5)2U]2(μ-H6:H6-C6H6), Synthesized via a New Mode of (C5Me5)3M Reactivity. *J. Am. Chem. Soc.* **2004**, *126* (44), 14533–14547. https://doi.org/10.1021/ja0463886.

(4) Bain, G. A.; Berry, J. F. Diamagnetic Corrections and Pascal's Constants. *J. Chem. Educ.* **2008**, *85* (4), 532. https://doi.org/10.1021/ed085p532.

(5) Dolomanov, O. V; Bourhis, L. J.; Gildea, R. J.; Howard, J. A. K.; Puschmann, H. OLEX2: A Complete Structure Solution, Refinement and Analysis Program. *J. Appl. Crystallogr.* **2009**, *42* (2), 339–341. https://doi.org/10.1107/S0021889808042726.

(6) Sheldrick, G. M. A Short History of SHELX. *Acta Crystallogr. Sect. A* **2008**, *64* (1), 112–122. https://doi.org/10.1107/S0108767307043930.

(7) Sheldrick, G. M. Crystal Structure Refinement with SHELXL. *Acta Crystallogr. Sect. C* **2015**, *71* (1), 3–8. https://doi.org/10.1107/S2053229614024218.

(8) *ADF 2020.101*, SCM, Theoretical Chemistry, Vrije Universiteit, Amsterdam, The Netherlands, http://www.scm.com. E. J. Baerends, T. Ziegler, A. J. Atkins, J. Autschbach, O. Baseggio, D. Bashford, A. Bérces, F. M. Bickelhaupt, C. Bo, P. M. Boerrigter, C. Cappelli, L. Cavallo, C. Daul, D. P. Chong, D. V. Chulhai, L. Deng, R. M. Dickson, J. M. Dieterich, F. Egidi, D. E. Ellis, M. van Faassen, L. Fan, T. H. Fischer, A. Förster, C. Fonseca Guerra, M. Franchini, A. Ghysels, A. Giammona, S. J. A. van Gisbergen, A. Goez, A. W. Götz, J. A. Groeneveld, O. V. Gritsenko, M. Grüning, S. Gusarov, F. E. Harris, P. van den Hoek, Z. Hu, C. R. Jacob, H. Jacobsen, L. Jensen, L. Joubert, J. W. Kaminski, G. van Kessel, C. König, F. Kootstra, A. Kovalenko, M. V. Krykunov, P. Lafiosca, E. van Lenthe, D. A. McCormack, M. Medves, A. Michalak, M. Mitoraj, S. M. Morton, J. Neugebauer, V. P. Nicu, L. Noodleman, V. P. Osinga, S. Patchkovskii, M. Pavanello, C. A. Peeples, P. H. T. Philipsen, D. Post, C. C. Pye, H. Ramanantoanina, P. Ramos, W. Ravenek, M. Reimann, J. I. Rodríguez, P. Ros, R. Rüger, P. R. T. Schipper, D. Schlüns, H. van Schoot, G. Schreckenbach, J. S. Seldenthuis, M. Seth, J.G. Snijders, M. Solà, M. Stener, M. Swart, D. Swerhone, V. Tognetti, G. te Velde, P. Vernooijs, L. Versluis, L. Visscher, O. Visser, F. Wang, T. A. Wesolowski, E. M. van Wezenbeek, G. Wiesenekker, S. K. Wolff, T. K. Woo, A. L. Yakovlev.

(9) G. te Velde, F. M. Bickelhaupt, E. J. Baerends, C. Fonseca Guerra, S. J. A. van Gisbergen, J. G. Snijders, T. Ziegler, Chemistry with ADF. *J. Comput. Chem*. **22**, 931–967 (2001).

(10) C. Fonseca Guerra, J. G. Snijders, G. te Velde, E. J. Baerends, Towards an order-N DFT method. *Theor. Chem. Acc*. **99**, 391–403 (1998).

(11) *AMS 2020.101*, SCM, Theoretical Chemistry, Vrije Universiteit, Amsterdam, The Netherlands, http://www.scm.com. R. Rüger, M. Franchini, T. Trnka, A. Yakovlev, E. van Lenthe, P. Philipsen, T. van Vuren, B. Klumpers, T. Soini

(12) J. P. Perdew, K. Burke, M. Ernzerhof, Generalized Gradient Approximation Made Simple. *Phys. Rev. Lett*. **77**, 3865–3868 (1996).

(13) J. P. Perdew, K. Burke, M. Ernzerhof, Generalized Gradient Approximation Made Simple. *Phys. Rev. Lett*. **78**, 1396 (1997).

(14) S. Grimme, J. Antony, S. Ehrlich, H. Krieg, A consistent and accurate ab initio parametrization of density functional dispersion correction (DFT-D) for the 94 elements H-Pu. *J. Chem. Phys.* *132*, 154104 (2010).
(15) S. Grimme, S. Ehrlich, L. Goerigk, Effect of the damping function in dispersion corrected density functional theory. *J. Comput. Chem*. **2011**, *32*, 1456–1465.
(16) M. Ernzerhof, G. E. Scuseria. Assessment and validation of a screened Coulomb hybrid density functional. *J. Chem. Phys*. **1999**, *119*, 5029–5036.
(17) C. Adamo, V. Barone. Toward reliable density functional methods without adjustable parameters: The PBE0 model. *J. Chem. Phys*. **1999**, *110*, 6158–6170.
(18) Y. Akinaga, S. Ten-no. Range-separation by the Yukawa potential in long-range corrected density functional theory with Gaussian-type basis functions. *Chem. Phys. Lett*. **2008**, *462*, 348–351
(19) E. van Lenthe, E. J. Baerends, J. G. Snijders, Relativistic regular two-component Hamiltonians. *J. Chem. Phys*. **1993**, *99*, 4597–4610.
(20) E. van Lenthe, E. J. Baerends, J. G. Snijders, Relativistic total energy using regular approximations. *J. Chem. Phys*. **1994**, *101*, 9783–9792.
(21) E. van Lenthe, R. van Leeuwen, E. J. Baerends, J. G. Snijders, Relativistic regular two-component Hamiltonians. *Int. J. Quantum Chem*. **1996**, *57*, 281–293.
(22) E. van Lenthe, E. J. Baerends, Optimized Slater-type basis sets for the elements 1–118. *J. Comput. Chem*. **2003**, *24*, 1142–1156.
